# IoT in medical diagnosis: detecting excretory functional disorders for Older adults via bathroom activity change using unobtrusive IoT technology

**DOI:** 10.3389/fpubh.2023.1161943

**Published:** 2023-09-29

**Authors:** Bessam Abdulrazak, Hassan Mostafa Ahmed, Hamdi Aloulou, Mounir Mokhtari, F. Guillaume Blanchet

**Affiliations:** ^1^AMI-Lab, Université de Sherbrooke, Sherbrooke, QC, Canada; ^2^ReDCAD, Centre de Recherche en Numérique de Sfax, Sakiet Ezzit, Tunisia; ^3^Institute Mine-Telecom, Paris, France; ^4^Département de biologie, Faculté des sciences, Université de Sherbrooke, Sherbrooke, QC, Canada; ^5^Département de mathématiques, Faculté des sciences, Université de Sherbrooke, Sherbrooke, QC, Canada; ^6^Département des sciences de la santé communautaire, Faculté de médecine et des sciences de la santé, Université de Sherbrooke, Sherbrooke, QC, Canada

**Keywords:** Internet of Things, excretory functional disorders, early detection, irritable bowel syndrome, urinary tract infection, older adults

## Abstract

The Internet of Things (IoT) and Artificial Intelligence (AI) are promising technologies that can help make the health system more efficient, which concurrently can be particularly useful to help maintain a high quality of life for older adults, especially in light of healthcare staff shortage. Many health issues are challenging to manage both by healthcare staff and policymakers. They have a negative impact on older adults and their families and are an economic burden to societies around the world. This situation is particularly critical for older adults, a population highly vulnerable to diseases that needs more consideration and care. It is, therefore, crucial to improve diagnostic and management as well as proposed prevention strategies to enhance the health and quality of life of older adults. In this study, we focus on detecting symptoms in early stages of diseases to prevent the deterioration of older adults' health and avoid complications. We focus on digestive and urinary system disorders [mainly the Urinary Tract Infection (UTI) and the Irritable Bowel Syndrome (IBS)] that are known to affect older adult populations and that are detrimental to their health and quality of life. Our proposed approach relies on unobtrusive IoT and change point detections algorithms to help follow older adults' health status daily. The approach monitors long-term behavior changes and detects possible changes in older adults' behavior suggesting early onsets or symptoms of IBS and UTI. We validated our approach with medical staff reports and IoT data collected in the residence of 16 different older adults during periods ranging from several months to a few years. Results are showing that our proposed approach can detect changes associated to symptoms of UTI and IBS, which were confirmed with observations and testimonies from the medical staff.

## 1. Introduction

Early detection and prevention of health issues are today's major public health challenges facing medical staff and policymakers (1, 2). This situation is more critical for older adults since aging is associated with a serious decline in physical and cognitive abilities, a situation that is emphasized by the poor management of aging-related health problems. Nowadays, existing geriatric services have limited abilities to detect possible health changes and adapt medical assessment and intervention for older adults. Bridging the gap between these geriatric needs and existing services is a major incentive to improve the impact of these services. We argue that detecting older adults' possible health problems as early as possible helps reduce the economic burden on society, improve quality of life for older adults, and promote healthy aging (3, 4).

Technological observations based on the Internet of Things (IoT) and artificial intelligence (AI) are perceived as possible solutions for continuous monitoring and early detection of older adults' health problems. They enable us to collect real-time data and make prompt decisions that help medical staff (e.g., geriatricians) detect health-related problems at an early stage, without the need to perform classical tests (e.g., psycho-geriatric tests) that have limitations including assessment inaccuracies and the difficulty for older adults to recall past events. Therefore, we propose an unobtrusive approach for long-term behavior monitoring and early detection of possible changes in older adults' health status based on IoT and change point detection analysis. These technologies enrich medical observations and enhance medical assessment by providing new objective observations of daily living activities. Additionally, classical methods are insufficient to follow health status daily (5) because they retrospectively approach changes after their occurrences and do not focus on the early detection of changes. In this study, we focused on the early detection of digestive and urinary system disorders in older adults. Approximately 10%−15% of older adults are estimated to develop irritable bowel syndrome (IBS) in developed countries (6), numbers that can increase to 45% in underdeveloped countries (7). As for urinary tract infections (UTIs), ~2% of the world's population develops this condition every year (8).

Our approach proposes a way to detect changes in a subject's bathroom activity behavior. However, our method does not assess the cause underlying this behavior change. It was only following the medical staff's assessment of our results that we were able to associate our results with a UTI.

## 2. Background

The human excretory system is responsible for removing excess undesirable material, usually fluids and solids, from the body to ensure the body continues to work efficiently. The human excretory system is composed of multiple subsystems, each of which is responsible for getting rid of a particular form of body waste. The urinary and digestive systems are subsystems that are part of the excretory system. The urinary system is responsible for filtering blood and excreting excess water and salts as urine (9). As for the digestive system, it is responsible for getting rid of indigestible food particles in the form of solid feces through defecation (10). IBS and UTIs are among the known problems of the human excretory system.

IBS is a disorder that can occur in the large intestines, a part of the digestive system. The most common symptoms associated with IBS include abdominal pain, usually associated with bowel movements, nighttime diarrhea, and constipation. IBS usually occurs in people 50 years old or older (11).

UTI is a disorder that occurs in the urinary system. Symptoms may include sudden changes in urinary habits (such as increased frequency or urgency), pain or burning while urinating, and pain or tenderness in the pelvis, lower back, or abdomen (12). Untreated UTIs can spread from the bladder to the kidneys and beyond. As such, treating a UTI early can keep it from spreading and overwhelming the immune system, especially in older adults or anyone with a deficient immune system (13). UTI is one of the most commonly diagnosed infections in older adults. It is the most frequently diagnosed infection in long-term care residents, accounting for over a third of all nursing home-associated infections (14, 15). It is second only to respiratory infections in hospitalized patients and community-dwelling adults over the age of 65 (16, 17).

As our population ages, the burden of IBS and UTI in older adults is expected to grow simply because there is an increased number of older adults in the population, resulting in the need to improve diagnostic, management, and prevention strategies; elements that are critical to enhancing the health of older adults. As a first step to accomplishing this goal, we need to improve the follow-up of the overall bathroom activity of older adults to gain a better understanding of their general bathroom behavior. Hence, in the present study, we propose an unobtrusive IoT system to monitor the overall bathroom behavior of older adults inside their residences, which will enable us to detect unusual emerging behaviors across their overall bathroom behavior.

## 3. Related work

There are multiple scales and tests (e.g., psycho-geriatric) in medical science that analyze older adults' behavior and detect possible health changes. Among these scales, the Short Emergency Geriatric Assessment (SEGA) evaluates the frailty of older adults (18), the Mini-Mental State Examination (MMSE) targets cognitive changes, such as orientation problems, attention difficulties, and language troubles (19), the Geriatric Depression Scale (GDS) investigates changes in mood and emotions; e.g., sadness, sedentary life, and depression (20), the Instrumental Activities of Daily Living (IADL) identifies changes in activities of daily living associated with autonomy loss (21), and the Mini Nutritional Assessment (MNA) investigates nutritional changes, such as eating difficulties, weight loss, and protein intake insufficiency (22).

While these medical scales and tests help medical staff (e.g., geriatricians) analyze and evaluate older adults' behavior and identify possible health problems, there are limits to their efficiency. For example, it is inconvenient for older adults to recall past events in full detail at the time of an assessment (23). Therefore, subjective information and missing details might influence assessment results (24). Often, it is also impractical for older adults to move to a specific location to get an assessment (25). In addition, the assessments require older adults to reply to a given set of questions or perform specific tasks, which may have a negative impact, such as anxiety, if they are unable to reply to some questions or perform some tasks. Therefore, medical staff would benefit from using additional means to gather more thorough and complete objective observations to complete their medical assessment. In this respect, IoT monitoring technologies can be used to closely follow older adults in their living environment (e.g., at home), to detect possible health changes early (26).

IoT technologies offer portable devices, and medical equipment that can be used to gather data on patients, identify diseases, keep track of the patient's health, and send out notifications once a medical emergency is detected. These solutions include a variety of technologies, such as electrocardiogram (ECG) and pneumography monitoring systems (27–29), glucose level monitoring solutions (30, 31), temperature monitoring solutions (32, 33), and blood pressure monitoring systems (34–36). IoT technology can be used to detect short-term health problems, e.g., Aloulou et al. (37) deployed movement, pressure, proximity, and vibration sensors in nursing home rooms to detect night wandering and toilet falls. Semantic reasoning rules recognize the activities of daily living in real time and notify formal caregivers about potential anomalies. Rantz et al. (38) used motion and bed sensors to track movement, pulse, breathing, and bed restlessness to retrospectively detect health changes. Following the occurrence of significant health events, geriatricians review the monitoring data to investigate possible correlations. Another study (39) focused on sleep monitoring, as a lack of sleep may increase the risk of cognitive decline in older adults. A remote and non-intrusive technology was proposed to help patients monitor their sleep at home. A sensor mat equipped with an integrated micro-bending multimode fiber was deployed and evaluated in a free-living environment. The study enabled researchers to analyze the participants' sleep quality using various parameters deduced from the sensor mat. Vital signs, namely heart rate, respiratory rate, and body movements, were also reported to detect abnormal sleep patterns. Sensory observations have helped care-focused medical staff focus on areas that require more detailed attention, confirm their medical assessments, and detect patterns of decline not usually detected during regular office visits. A complete technical review for sleep cycle monitoring is conducted by the same authors afterward in Siyanbade et al. (40).

Based on AI technologies, other solutions were proposed to detect mental health problems, depression, stress, and bipolar disorder. In the study proposed by Alam et al. (41), a convolutional neural network (CNN) was used to analyze and classify a person's mood into six different categories: happy, thrilled, sad, calm, distressed, and angry. An advanced machine learning system was also adopted by Pandey (42) to identify stress periods in advance using heart rate. The proposed solution informs the patient about their stress level and prevents accidents. Similarly, Zekri et al. (43) were interested in detecting older adults' behavior changes. In their study, they modeled older adults' behavior and defined the normal behavior of a person as a sequence of four activities (sleeping, eating, taking a shower, and leaving home). An unsupervised approach based on the density-based clustering (DBSCAN) algorithm was applied, and the deviations were detected by computing a similarity score between the current behavior of the older adult and her/his normal behavioral pattern. Another study (44) applied deep learning methods, including CNNs and extreme learning machines (ELMs), to differentiate between ballistocardiogram (BCG) and non-BCG signals. BCG signals were collected using an IoT-based micro-bend fiber optic sensor mat from 10 patients with obstructive sleep apnea. The study used three methods to balance the number of BCG and non-BCG signals, including undersampling, oversampling, and generative adversarial networks (GANs). The system's performance was evaluated using 10-fold cross-validation, and the best results were obtained using CNN-ELM with GANs as the data balancing method. The results showed an average accuracy, precision, recall, and F-score of 94%, 90%, 98%, and 94%, respectively. Furthermore, the study presented by Sadek et al. (45) focused on contactless monitoring of heart rate (HR) using under-mattress (BCG) sensors. The authors studied the potential of two wavelet-based methods, the multiresolution analysis of the maximal overlap discrete wavelet transform (MODWT-MRA) and continuous wavelet transform (CWT), for HR detection using a microbend fiber optic sensor (MFOS). BCG signals were collected from 10 sleep apnea patients during an overnight polysomnography (PSG) study, and the MFOS was placed under the bed mattress. The PSG electrocardiogram (ECG) signals were used as a reference to evaluate the proposed HR detection algorithms. The results showed that the CWT with a derivative of Gaussian (Gaus2) provided slightly better results compared to the MODWT-MRA, CWT (frequency B-spline), and CWT (Shannon). However, the total precision for MODWT-MRA was higher than Gaus2.

As previously detailed above, IoT sensor observations can help care-focused medical staff in their practice. However, we could not find other technological solutions in the literature that help match observations with UTI and IBS symptoms. In this field, the gold standard to diagnose UTI and IBS includes (1), for UTI, lab screening through urine sample analysis, and (2) for IBS, stool tests. Moreover, healthcare providers are likely to evaluate IBS with a complete medical history and a physical exam and try to match symptoms with the IBS definition. They may also run tests to rule out some other possible causes of these symptoms. In our approach, we argue that an IoT system can be used as an aiding technological tool to follow-up on health status (including UTI and IBS) by following subject activity, in this research, in the bathroom.

In our research, an IoT system, which encompasses motion sensors to detect the participant's movement inside the bathroom, is used along with conventional statistical methods to monitor and detect the change in the overall bathroom behavior of individual participants. As in our previous research, where we monitored and identified behavior change on a granular level, that is, activity (and sub-activity) levels were evaluated daily [e.g., (46–48)], this research focused on high-level behavior change detection. However, in the present study, the goal is to show that reporting triggers to healthcare professionals (based on behavior change detection), it enables them to be more efficient and offer better health follow-up. Moreover, we presented the ability to impute the missing data found along with the collected data at any missing rate. Furthermore, the level of analysis we propose here requires fewer sensors as well as less installation and maintenance costs. Put simply, the system we present here is more economically viable and can be used to monitor patients for long period with minimal cost. In addition, working at this high level requires less computation, since we do not need to follow/detect precise activities. In our case, we do not need to analyze the details of bathroom visits to trigger the alerts. If we analyze changes in the overall number of bathroom visits, we obtain the same result.

## 4. Methodology

Our methodology is based on three pillars: (1) real-time data collection in the subjects' environments, (2) adapted data analysis based on custom algorithms and visualization tools, and (3) results validation with caregivers.

### 4.1. Data collection

The data we used to illustrate our methodology were gathered through an infrastructure that covers all the IoT layers (49). The infrastructure (50) was deployed in the real-life studio residences of 16 older adults for periods ranging from several months to a few years in France (51). The subjects' codes, along with their gender, age class, and the monitoring duration, are presented in Table 1.

**Table 1:** Relevant information about participating subjects in the France experiment.

**Subject code**	**Gender**	**Age**	**Monitoring period**	
			**Start**	**End**
A	M	90–94	2014-09-23	2015-02-13
B	M	90–94	2015-03-15	2015-08-03
C	M	80–84	2015-09-10	2018-11-30
D	F	85–89	2014-09-23	2015-08-06
E	F	95–99	2015-08-13	2018-11-30
F	F	85–89	2014-09-23	2017-01-18
G	F	90–94	2014-09-23	2018-11-30
H	F	95–99	2014-09-14	2015-06-03
I	F	95–99	2015-06-04	2018-11-30
J	F	90–94	2014-11-24	2015-10-12
K	M	85–89	2016-10-11	2017-01-30
L	F	90–94	2017-04-04	2018-11-30
M	M	90–94	2016-10-11	2018-11-30
N	F	90–94	2016-10-11	2018-11-30
P	F	90–94	2017-04-07	2017-06-15
V	M	65–69	2017-10-20	2018-11-30

**Figure 1:**
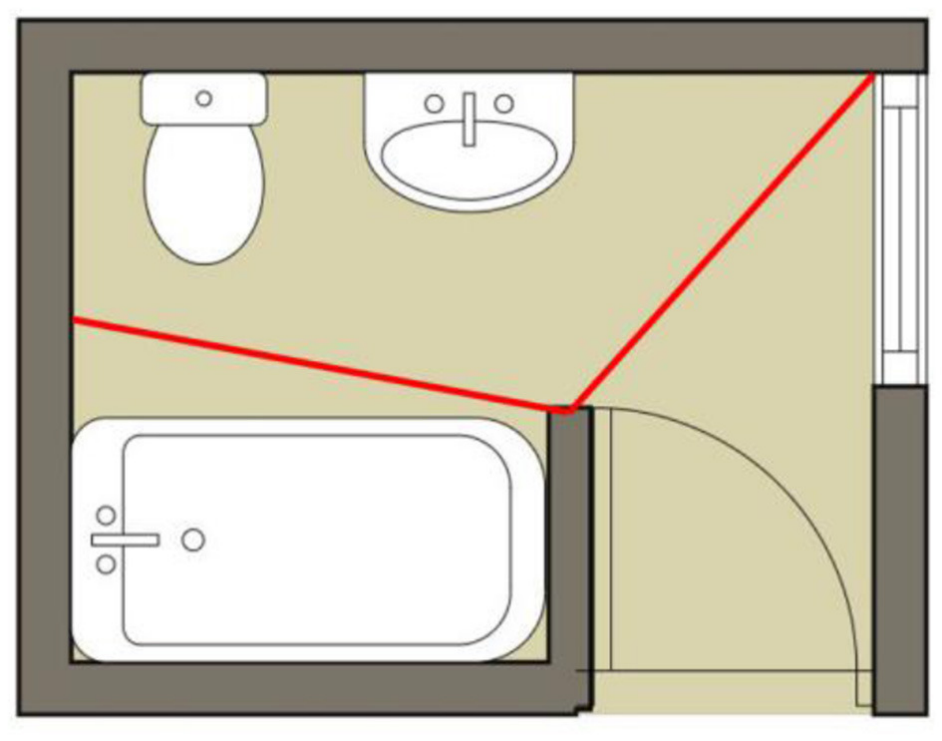
Schematic description of the bathroom with the spatial location of sensors.

Bathroom activity was monitored around the clock using an infrastructure mainly based on motion sensors mounted in the participants' bathrooms. These motion sensors are mounted so that they can capture motion over the sink and the toilet area of each bathroom. A schematic of the bathroom describing the spatial location of the sensor is presented in Figure 1. In each washroom, we used Wyze Sense motion sensors with a 120° field of view and a range of approximately 8 meters. These sensors are characterized by an adjustable relaxation time ranging from 5 s to 15 s.

**Figure 2:**
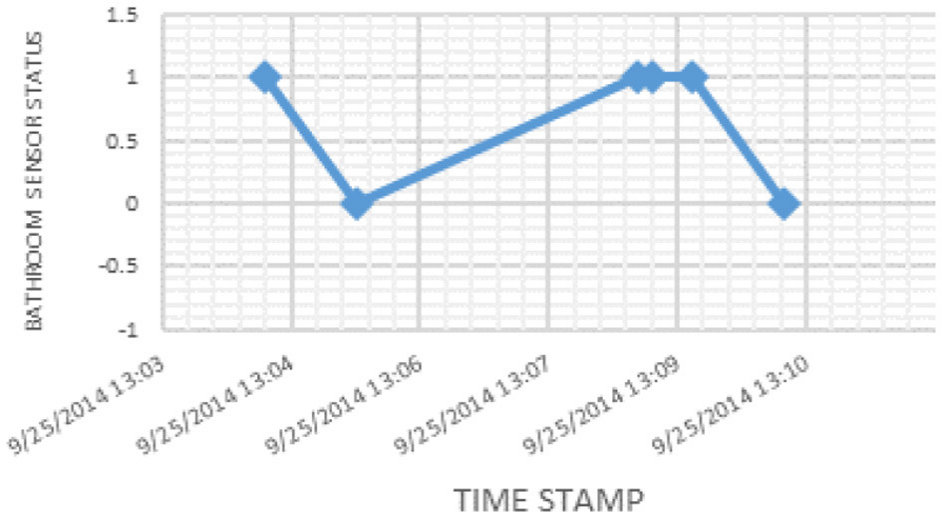
Sensor signal values for a snapshot of activity.

The presence of an older adult inside the bathroom is recorded by the sensor as a logic signal of 1, while the absence is recorded as a logic signal of 0. The corresponding sensor signal values for a snapshot of activity are presented in Figure 2. The details of the deployment experiment can be found in Kaddachi et al. (50, 52).

### 4.2. Data analysis

The proposed algorithm was first used to calculate overall daily bathroom activities and to define daily living patterns. From the overall daily activity, we thus focus on the whole objective excretory function. In this study, we use conventional mathematical methods to identify the change points across the time series.

A pre-processing algorithm was used to convert the raw logic signal data into an overall daily bathroom activity, while a post-processing algorithm was used to identify the timestamps at which behavior changes occurred. The pre-processing and post-processing algorithms are presented in Algorithms 1, 2, respectively.

**Algorithm 1:** Daily overall bathroom activity calculation.

**Algorithm 2:** Daily overall bathroom activity anomaly detection.

Prior to applying the pre-processing algorithm, we imputed missing data using our novel Bayesian Gaussian Approach (BGaP) (53). The missing data type found in our collected subjects' data was considered to be missing completely at random (MCAR), which assumes that the missingness of the data presents no correlations to any potential variables structuring the time series (53). The imputed subject's activity time series was then normalized to the complete number of hours per day, i.e., the resulting pre-processed bathroom activity time series represents the average number of bathroom visits per hour in a day. This is an essential step to compare the bathroom activity for all subjects together.

The two algorithms were used iteratively to identify and retrieve changes in activities in the overall daily bathroom activity and to identify the associated time at which such changes occur. Specifically, Algorithm 2 defines a Pruned Exact Linear Time (PELT) technique applied to the overall daily bathroom activity data that was used to identify the predicted change points. PELT technique is a method for detecting change points in a time series; it is an efficient and exact algorithm for change point detection, meaning that it provides an exact solution and is computationally efficient, making it well-suited for large datasets. It is also a “pruned” algorithm, which eliminates unnecessary calculations to improve computational efficiency. The PELT technique works by considering a sliding window of the time series and calculating a cost function that measures the difference between the current window and the previous window. If the cost function exceeds a certain threshold, a change point is identified. The process is repeated for each window in the time series, and the change points are identified as the points where the cost function exceeds the threshold. One advantage of the PELT technique is its ability to handle multiple change points in the time series, making it well suited for datasets with complex patterns. Additionally, the algorithm can easily be adapted to different types of cost functions, allowing for the detection of different types of change points.

### 4.3. Validation

The validation of our results was based on observations from collaborating medical staff (doctors, caregivers, and nurses), as well as the feedback from our iterative meetings. We have collaborated with two caregivers from a nursing home and one geriatrician for individual participants.

The caregivers recorded all significant observations daily and continuously collected electronic health records. Individual participants have monitored via nurse visits. These nurses' visits monitored participants a few times per day for medicine taking, toilet entry assistance, room cleaning, and nutritional services. Nurses reported all interventions, formal and informal observations, special health events, and social habits in developed software. These electronic health records include geriatric assessment results, hospitalizations, treatments, and physical, emotional, and cognitive problems.

In addition, regular review meetings allowed for accurate investigation of possible causes of detected changes and any health problems associated with them. These meetings were planned with older adults, family members, and family doctors (each for 4 months with a nursing home and each for 2 months with individual houses). Medical staff evaluated the medical relevance of investigated change explications by reviewing all past detected changes and correlating them with medical records (e.g., geriatric scales, cognitive diagnosis, and prescribed treatments).

## 5. Results and discussion

Following, we present the results of our algorithms applied to the bathroom activities of the different participants from two different perspectives.

- First, we present the time series of the bathroom activity after imputation for the entire monitoring period for each participant, where we highlight the time of the detected changes as a result of the PELT algorithm by a vertical red line (Figures 3–9 and Supplementary Figures 1–9), each vertical red line along the overall bathroom activity time series represents a change in the participant's bathroom behavior in comparison with the preceding window of calculation, where this change represents either an increase or a decrease in the number of the subject's bathroom visits. *We claim that changes caused by an increase in bathroom visits are due to the participant experiencing an UTI*.
- Second, we presented a heatmap illustrating the entire bathroom activity for each subject day by day. This representation helped the medical staff compare the subject's bathroom activity across different months of the year and, hence, during the entire monitoring period. In addition, with the aid of the heatmap (Figures 10–15 and Supplementary Figures 10–19) the physician can easily pinpoint the day when the highest number of bathroom visits occurred, leading to a feasible identification of the UTI.

**Figure 3:**
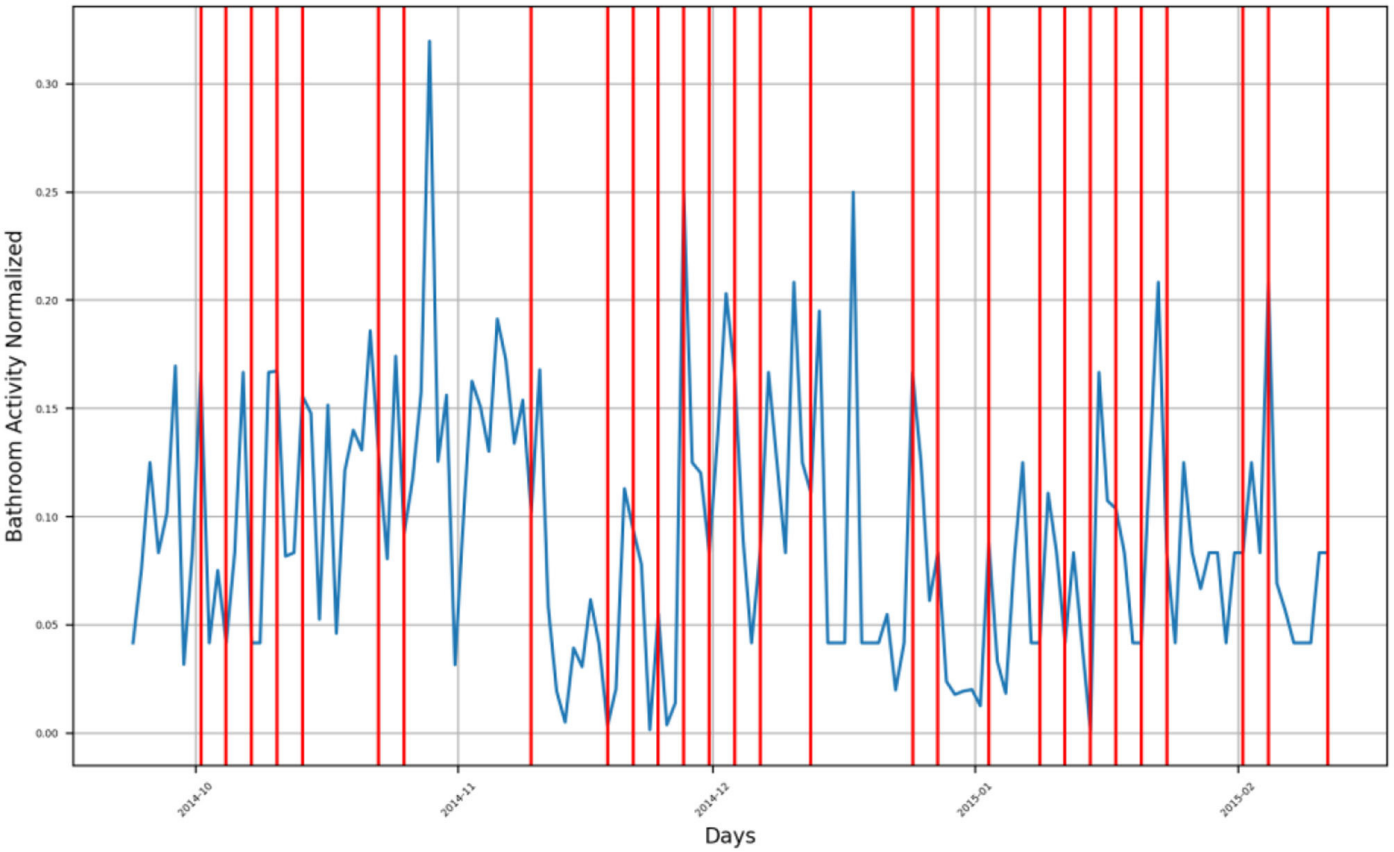
Bathroom activity time series for subject A, with detected change time stamps overlapped as vertical lines.

It is worth mentioning that the heatmaps are shown without imputation, while the bathroom activity time series are presented after imputing the missing data, in order to prevent misleading visual results by the physicians.

### 5.1. Time series representation

For subject A, the bathroom activity of the monitoring period extended from late September 2014 to the beginning of February 2015, where we can notice that the subject has four (4) groups of bathroom activity behavior change, as presented in Figure 3.

- First group is found in the second half of October 2014, when the subject experienced a behavior change as an increase in bathroom visits at the end of the first half of October. After this, the subject had a second behavior change, which presented itself as a decrease in his bathroom visits, followed by a third change presenting an increase in bathroom visits. The fourth change is composed of another increase in bathroom visits, and another change on the first of November was also due to an increase in bathroom visits.
- Second group of changes is found in mid-November 2014, showing a positive inflection point of the subject's bathroom activity, meaning that the subject had an increasing behavior of bathroom activity followed by a decreasing behavior. This group consisted of three behavior change timestamps: the first one indicating increasing activities, and the other two indicating decreasing activities.
- Third group of changes is found in the first half of December 2014, where the first change, as well as the second one, indicates a decrease in bathroom visits, while the third one indicates an increase in bathroom visits.
- Fourth group of changes is found in the second half of December 2014 and during the first week of January 2015, where changes in timestamps indicate a decrease in bathroom visits. The last group is found in the second half of January 2015 and during the first week of February 2015, where changes indicate an increase in bathroom visits.

Both the second group and the third group form an envelope around a sharp increase in the subject's bathroom activity in the last week of November 2014. Similarly, both the third and fourth groups form an envelope around another sharp increase in the subject's bathroom activity in mid-December 2014.

**Figure 4:**
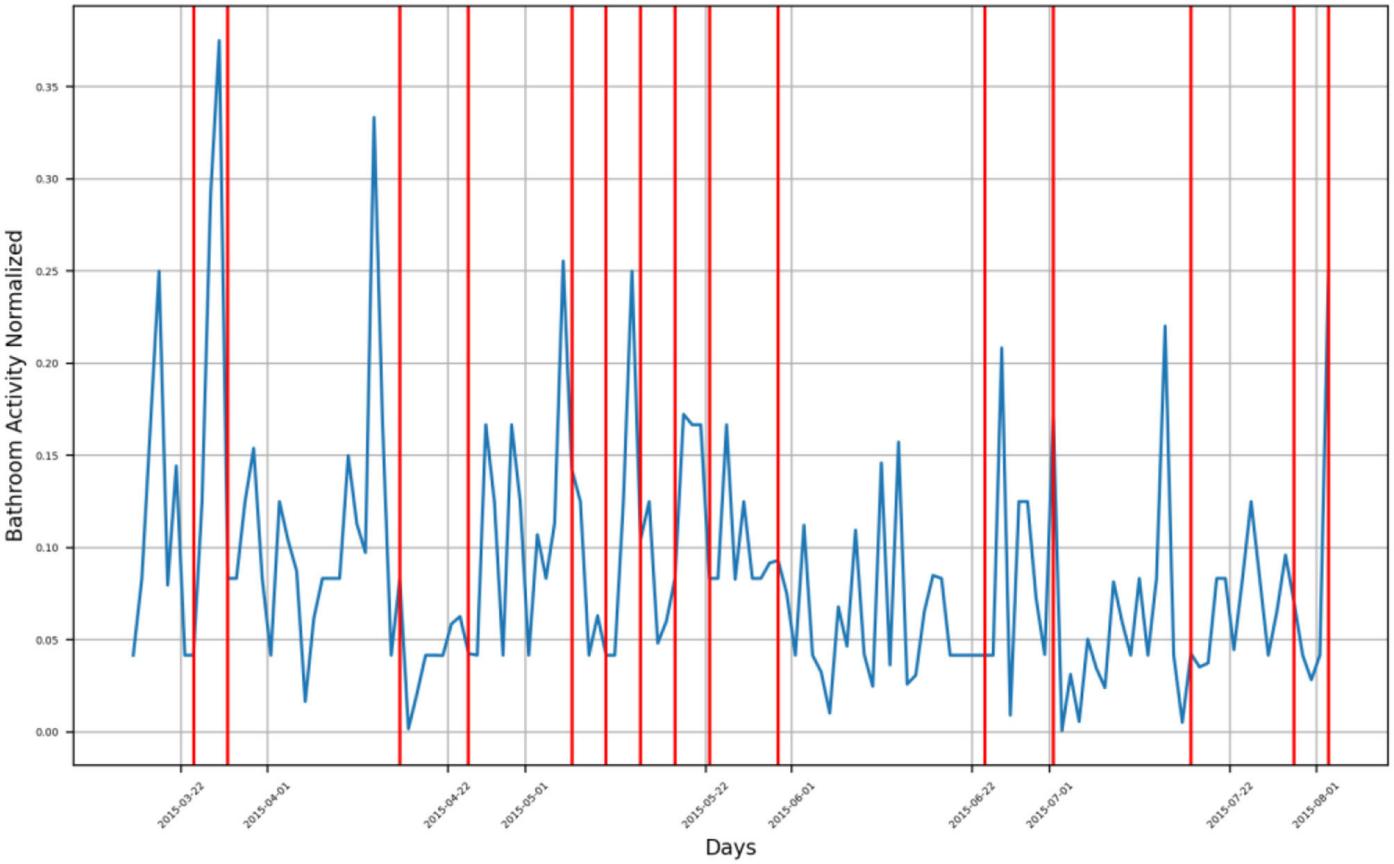
Bathroom activity time series for subject B, with detected change time stamps overlapped as vertical lines.

For subject B, the monitoring period extended from mid-March 2015 to the beginning of August 2015, where there are three groups of bathroom activity behavior changes, as presented in Figure 4.

- First group consists of four change timestamps around mid-April 2015, indicating a decrease in the subject's bathroom visits.
- Second group consists of six change timestamps spread along June 2015, where the first one indicates a decrease in the subject's bathroom visits, while both the second and the third one form an envelope around a changing timestamp indicating an increase in bathroom visits. The last three change timestamps indicate a decrease in bathroom visits.
- The last group consists of a single change timestamp indicating an increase in bathroom visits by the subject and is located on 2 August 2015.

Both the third group and the fourth group form an envelope around a sharp increase in the subject's B bathroom activity located in mid-December 2014.

**Figure 5:**
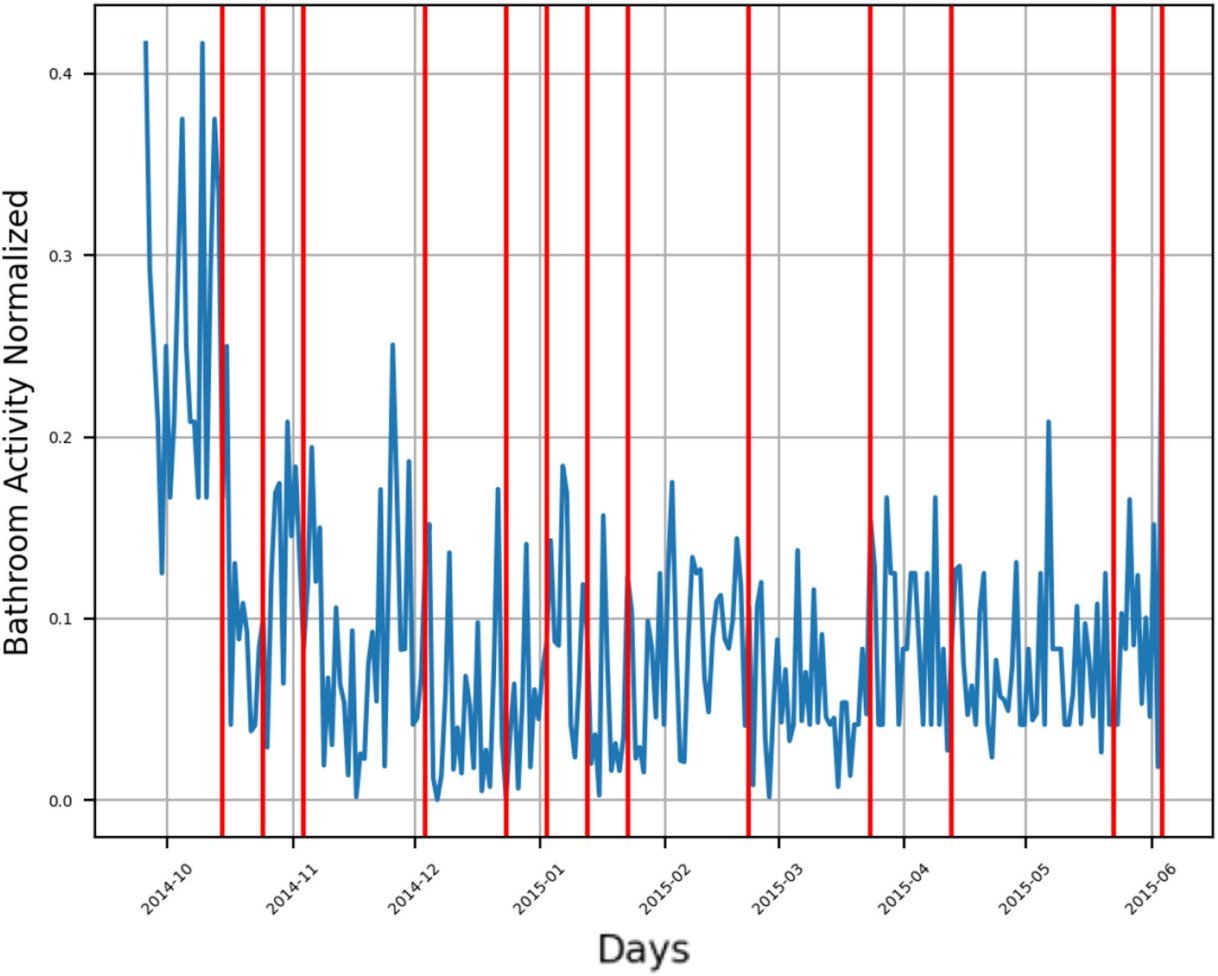
Bathroom activity time series for subject C, with detected change time stamps overlapped as vertical lines.

For Subject C, several groups of bathroom activity changed timestamps across the monitoring period, which extended from October 2015 to January 2018, as presented in Figure 5.

- The first group of behavior changes is located between October 2015 and January 2016, where bathroom activity behavior increased. In March 2016, a decreasing trend of bathroom visits was found, while another decrease in his bathroom visits was detected in May 2016. The subject's behavior started to increase again during the period extending from July 2016 to December 2017, where six detected behavior change timestamps occurred over this period, all of them representing the behavior change toward an increase in the subject's bathroom visits. A sudden decrease in the bathroom activity of the subject was detected in late January 2017, and another decrease in his bathroom activity was detected in March 2017 as well. Additional two-bathroom activity changes were detected in late May 2017 and on the first week of June 2017. A decreasing behavior in the subject's bathroom activity behavior was detected by three change timestamps between July 2017 and August 2017.
- Following, a period of increasing bathroom activity was detected by a group of change timestamps extending from October 2017 to November 2017, after which a sharp decrease in bathroom activity was detected in December 2017.
- A last group of change timestamps indicating a fluctuation in bathroom activity was found in January 2018.

**Figure 6:**
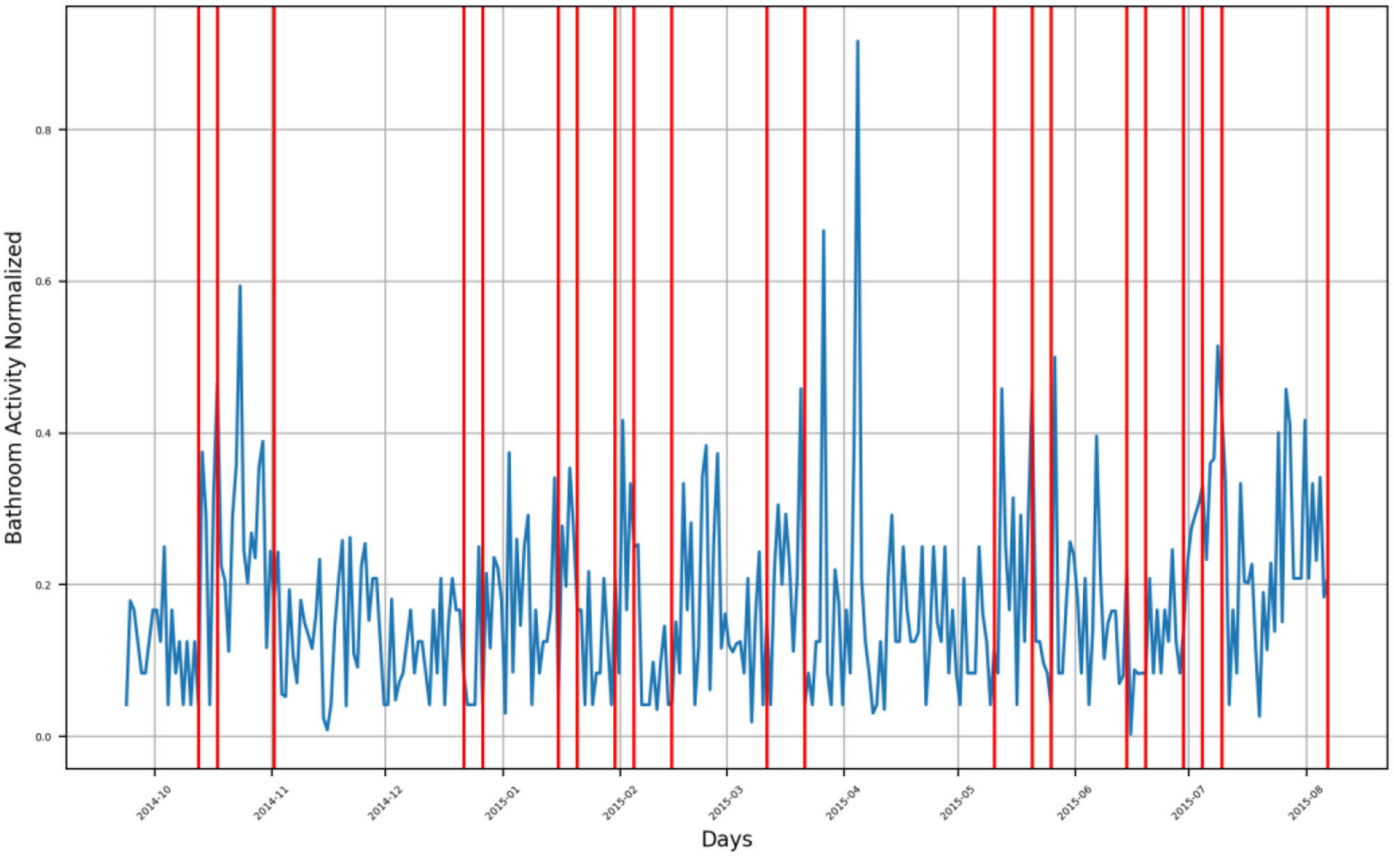
Bathroom activity time series for subject D, with detected change time stamps overlapped as vertical lines.

For subject D, the monitoring period extended from late September 2014 to August 2015, as presented in Figure 6. There are multiple groups of detected behavior change timestamps:

- First group includes a single change timestamp indicating a sudden increase in the number of bathroom visits in the first week of October 2014.
- Second group consists of three change timestamps representing a decrease in bathroom activity during November 2014, where the first timestamp represents a sharp decrease in bathroom activity, while the other two timestamps represent a slow decreasing trend of his bathroom activity.
- Third group consists of two change timestamps indicating a mild change in bathroom activity, one representing a mild decrease and the other representing a mild increase, both found in late December 2015.
- Fourth group consists of two change timestamps indicating an increase in bathroom activity were found in the first week of February 2015. A single change timestamp representing a decrease in bathroom activity is in the first week of March 2015.
- Fifth group consists of two change timestamps at the beginning of April 2015, representing a sharp increase in the subject's bathroom visits.
- Sixth group: a sudden increase in bathroom visits was found in mid-May 2015.
- Seventh group: the last group consists of two change timestamps indicating an increase in bathroom activity located in mid-July 2015 and at the beginning of August 2015.

**Figure 7:**
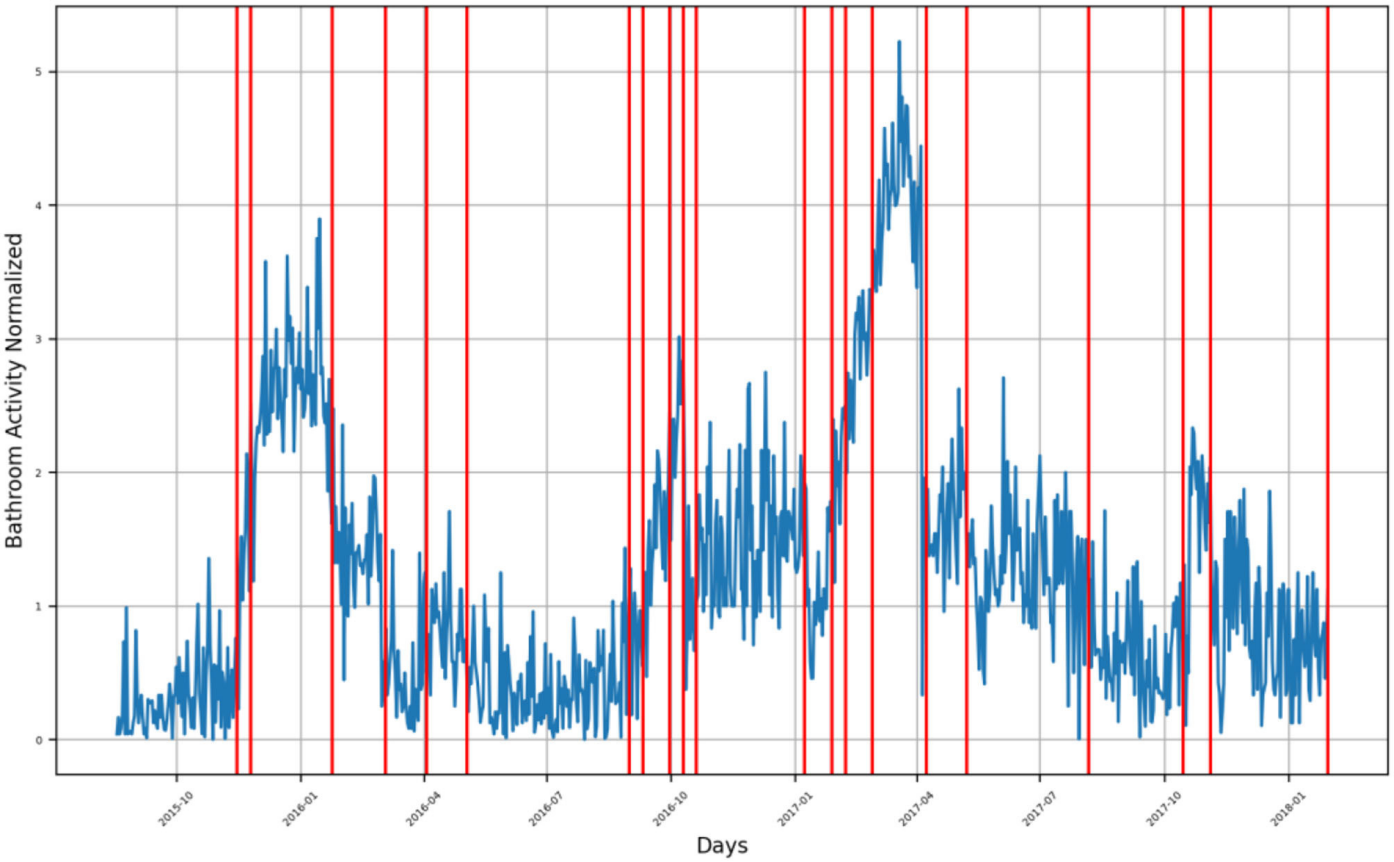
Bathroom activity time series for subject E, with detected change time stamps overlapped as vertical lines.

For subject E, bathroom activity was monitored from October 2015 to January 2018, as presented in Figure 7.

- First group: during the period extending from late September 2015 to late March 2016, the subject is showing an exponential growth trend in his bathroom activity behavior, within which there are two detected behavior change timestamps, first in late September 2015 and second in late December 2015. After this exponential growth period, there is a sharp decrease in the subject's bathroom activity in the last week of March 2016 that is detected by two change timestamps as an envelope around that week.
- Second group: exponential growth trends in the subject's bathroom activity were found between October 2016 and November 2016, where there are multiple detected change timestamps indicating an increase in activity.
- Third group: an increasing trend in bathroom activity was observed from February 2017 to May 2017, after which there was a mild decrease in the subject's bathroom activity until the end of the monitoring period in January 2018.

**Figure 8:**
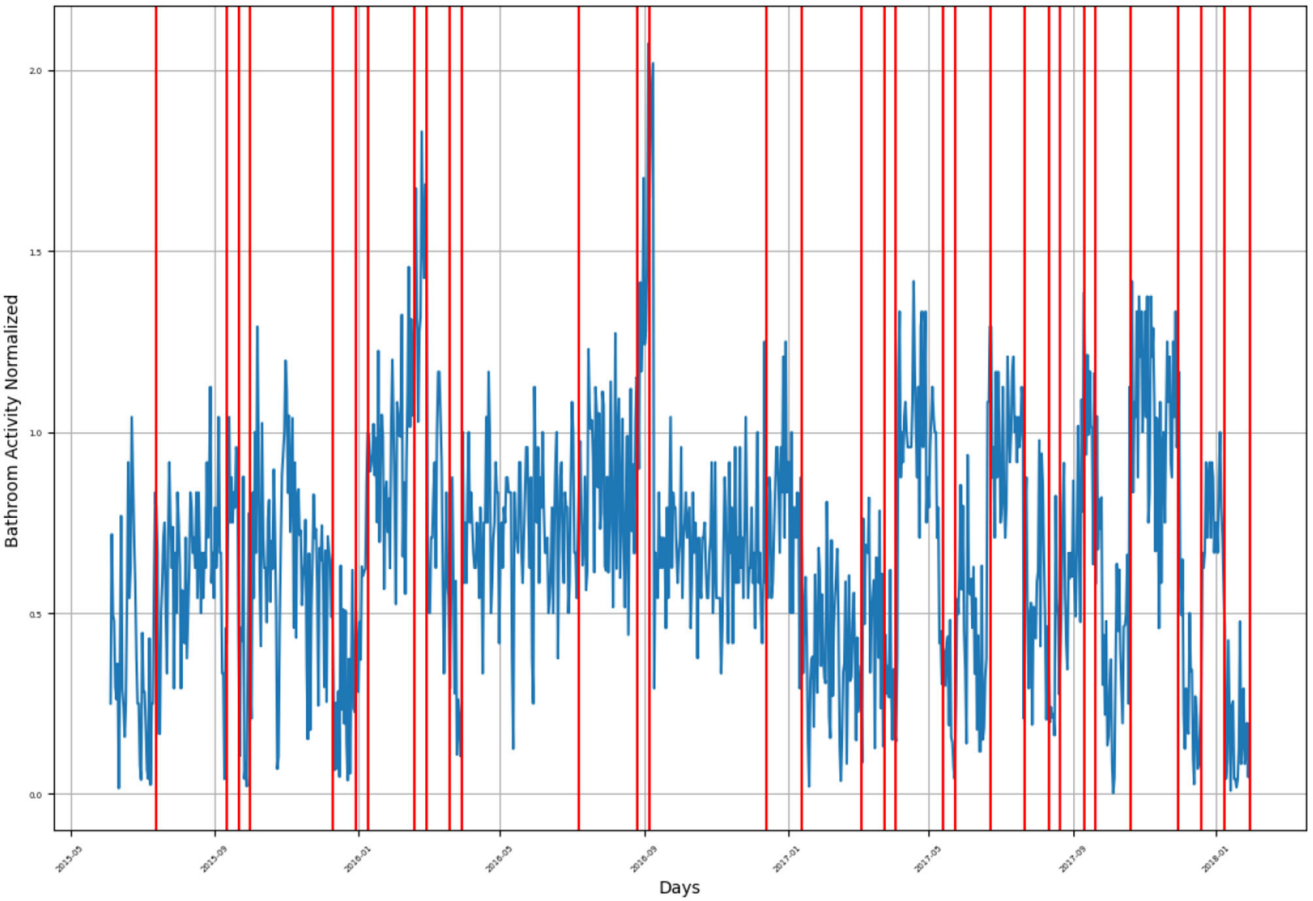
Bathroom activity time series for subject F, with detected change time stamps overlapped as vertical lines.

For subject F, bathroom activity was monitored from October 2014 to January 2017, as presented in Figure 8. During this period, there were three main segments of behavior,

- First segment extends from October 2014 to October 2015, during which the activity is neither following an increasing trend nor a decreasing trend; instead, multiple spikes are indicating a sharp increase in bathroom activity.
- Second segment extends from November 2015 to March 2016, where there is an exponential increase in the subject's bathroom activity followed by a decreasing trend extending from March 2016 to May 2016. There were multiple spikes of sharply increased bathroom visits that were also detected during this segment.
- Third segment: the last segment extends from June 2016 to the end of the monitoring period in January 2017. It represents an increase in the subject's bathroom activity.

**Figure 9:**
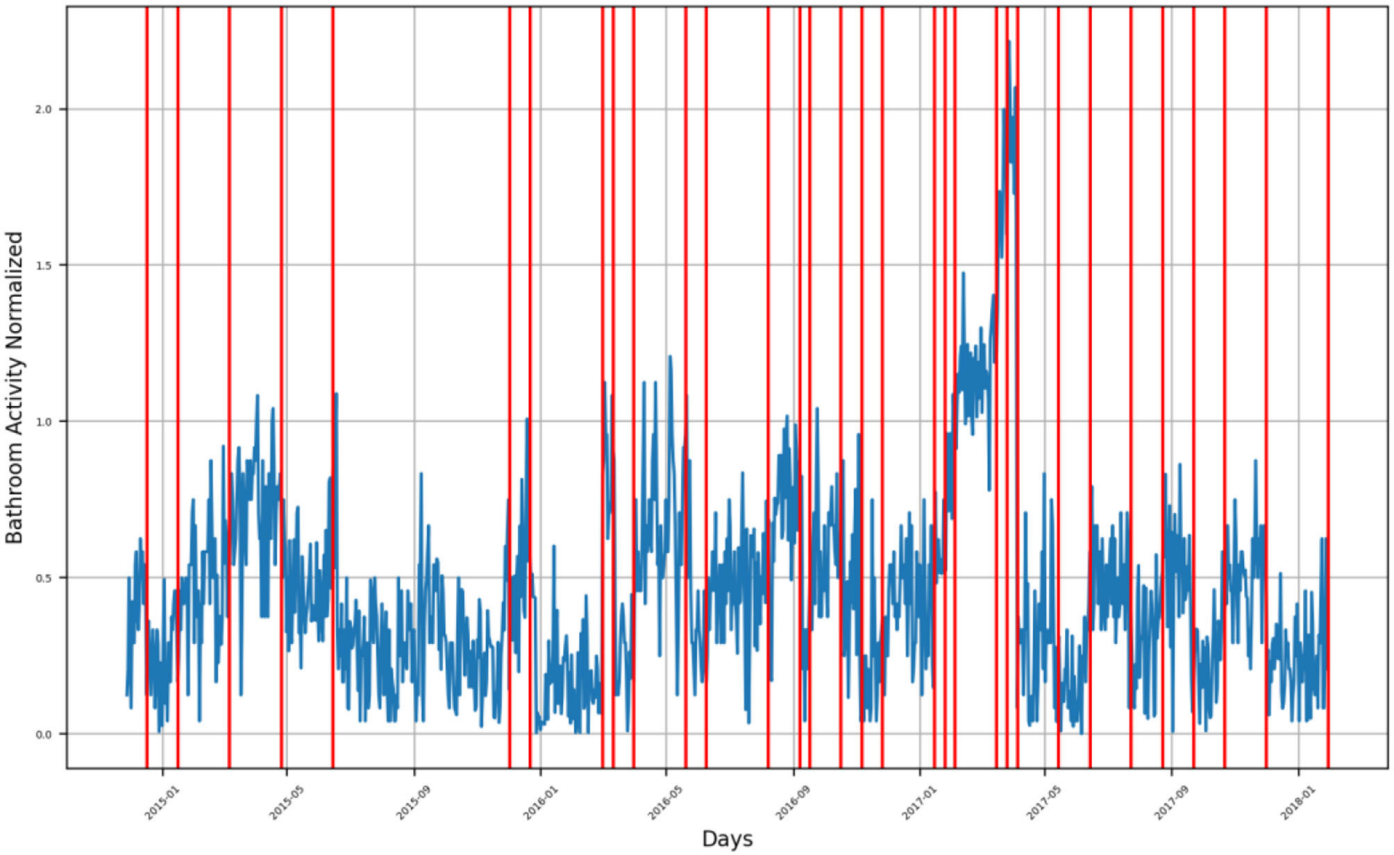
Bathroom activity time series for subject G, with detected change time stamps overlapped as vertical lines.

For subject G, the monitoring period extended from late December 2015 to January 2018, as presented in Figure 9, where the subject's bathroom activity behavior follows a cyclic pattern. There are multiple segments of behavior decrease:

- First segment was found in late December 2015.
- Second segment spanned from April 2015 to September 2015.
- Third segment was found in late October 2015.
- Fourth segment was found in late March 2016 and late May 2016.
- Fifth segment was found in September 2016 and from October 2016 to the end of November 2016.
- Sixth segment spans from December 2016 to March 2017.
- Seventh segment was found from May 2017 to September 2017 and from December 2017 to January 2018.

Other segments are representing an increasing bathroom activity behavior.

For subject H, the monitoring period extended from late September 2014 to June 2015, as presented in Supplementary Figure 1.

The general subject's behavior represented a decreasing bathroom activity behavior, among which there were multiple sharp increase in bathroom visits. Through the decreasing segment, there were two detected change timestamps in mid-October 2014 and early November 2014. During the few sharp increases in visits, there were six detected change timestamps in mid-December 2014, early January 2015, late March 2015, late April 2015, and early June 2015.

For subject I, the monitoring period extended from May 2015 to January 2018, as presented in Supplementary Figure 2. The subject's bathroom behavior represents cyclic behavior like that of the subject's **G**. The decreasing segments throughout the monitoring period are as follows:

- First segment represents a sharp decrease in bathroom activity compared to the surrounding normalized bathroom activity and was found in June 2015.
- Second segment represents a sharp decrease and was found in September 2015 and October 2015.
- Third segment represents a mild decrease in bathroom activity and extends from late October 2015 to March 2016.
- Fourth segment represents a mild decrease and spans from June 2015 to September 2015.
- Fifth segment represents a slow decrease and extends from January 2017 to April 2017.
- Sixth segment represents a sharp decrease in the subject's bathroom activity and spans from May 2017 to June 2017.
- Seventh segment represents a very sharp decrease in the subject's bathroom activity and was found at the beginning of December 2017.
- Eighth segment represents the last decreasing behavior segment found at the beginning of January 2018.

Otherwise, the subject was showing increasing behavior in his bathroom activity.

For subject F, bathroom activity was monitored from October 2014 until January 2017, as presented by Figure 8. During this period, there were 3 main segments of behavior,

- First segment extends from October 2014 until October 2015, during which the activity is neither following an increasing trend nor a decreasing trend, instead, there are multiple spikes indicating a sharp increase in bathroom activity.
- Second segment extends from November 2015 until March 2016, where there is an exponential increase in the subject's bathroom activity followed by a decreasing trend extending from March 2016 to May 2016. There were multiple spikes of sharply increased bathroom visits which were also detected during this segment.
- Third segment: The last segment extends from June 2016 until the end of the monitoring period in January 2017, it represents an increase in the subject's bathroom activity.

For subject J, the monitoring period extended from late November 2014 to October 2015, as presented in Supplementary Figure 3.

The detected behavior change timestamps indicating an increase in bathroom activity were found in mid-January 2015, at the beginning of February 2015, in late March 2015, in mid- and late May 2015, at the beginning of June 2015, and during the first half of September 2015. Otherwise, the remaining detected change timestamps represent decreasing bathroom activity behavior. Furthermore, the bathroom activity behavior of the subject showed a cyclic pattern starting in February 2015 and lasting until the end of the monitoring period.

For subject K, the monitoring period extended from October 2016 to February 2017, as presented in Supplementary Figure 4. There are only a few detected bathroom activity behavior change timestamps spread over the monitoring period:

- The detected change timestamp on 15 October 2016 represents a sharp decrease in the subject's activity, as does the timestamp in the second half of October 2016.
- During November 2016, all the detected change timestamps indicated a slow decrease in bathroom activity behavior. Whereas in December 2016, the detected change in timestamps indicated an increase in the subject's bathroom activity.
- Finally, the detected change in timestamps during January and February 2017 represent a decrease in the subject's bathroom activity.

For subject L, the monitoring period extended from April 2017 to February 2018, as presented in Supplementary Figure 5.

- The detected behavior change timestamps spanning from late April 2017 to the beginning of May 2017 indicate decreasing bathroom activity behavior, while behavior change timestamps in the second half of May 2017 indicate increased bathroom activity behavior.
- Furthermore, the single change timestamp in late June 2017 indicates an increase in bathroom activity behavior.
- During the period extending from July 2017 to mid-August, the general bathroom behavior of the subject was decreasing except for a few sharp spikes indicating a sudden increase in the behavior; the sharpest spike is at the end of July with a normalized value of 1.5 visits per hour.
- Another two decreasing behavior segments were found from late November 2017 to mid-December 2017 and from the beginning of January 2018 to mid-January 2018.

The detected behavior change timestamps along these two segments indicate a sharp and unexpected increase in the subject's bathroom activity behavior.

For subject M, the monitoring period extended from October 2016 to January 2018, as presented in Supplementary Figure 6. There are multiple groups of detected change timestamps, as follows:

- The first group extends from mid-October 2016 to November 2016, representing a positive inflection point in the subject's bathroom activity behavior, meaning that there has been a sudden increase followed by a sudden decrease in his activity behavior.
- During December 2016, there was a detected increase in the subject's bathroom behavior, while at the beginning of January 2017, there were two detected timestamps indicating a sudden decrease in the subject's bathroom behavior.
- At the beginning of March 2017, there was a detected decrease in the subject's behavior, while in April 2017, there were three detected change timestamps indicating an increase in the subject's behavior.
- From May 2017 to June 2017, there was a detectable decrease followed by an increase in the subject's bathroom behavior.
- In the first week of July 2017, there was a decrease in activity detected by two change timestamps.
- In late August 2017, another decrease in the subject's behavior was detected, followed by a mild decrease at the beginning of September 2017.
- Starting in October 2017, all the detected change timestamps indicate an increase in bathroom activity.

For subject N, the monitoring period extended from late October 2016 to January 2018, as presented in Supplementary Figure 7.

- The general bathroom activity behavior of the subject shows a slowly increasing pattern until July 2017, followed by a sudden decrease during July 2017, after which there was another slowly increasing behavior.
- The detected change timestamps in late October 2016 and at the beginning of November 2016 indicate decreasing bathroom behavior.
- In the beginning of December 2016, a sudden increase in activity was detected, followed by a sudden decrease in the subject's behavior.
- The detected change timestamps during the period extending from January 2017 to July 2017 indicate an increasing behavioral change for the subject.

For subject P, the monitoring period extended from late March 2017 to July 2017, as presented in Supplementary Figure 8. The general bathroom activity behavior of the subject shows a decreasing behavior at the beginning of the monitoring period, followed by increasing activity toward the end.

- First group: Two detected change timestamps indicate a decrease in the behavior, while the third one indicates an increase in the same behavior.
- In the first week of May 2017, there was a detected decrease in the subject's bathroom activity, while in the second half of May 2017, there was a detected increase in activity.
- At the end of the first week of June 2017 and on May 15, there were two detected change timestamps indicating an increase in the subject's activity behavior, whereas in the second half of May 2017, there was a detected timestamp that indicated a sudden increase in the subject's behavior.

For subject V, the monitoring period extended from October 2017 to February 2018, as presented in Supplementary Figure 9. There are four groups of detected behavior change timestamps along this period, as follows:

- The first group is found from late October 2017 until the beginning of November 2017 and consists of three detected timestamps, all indicating decreasing bathroom behavior for the subject.
- The second group consists of two detected timestamps, indicating a sudden increase in bathroom behavior after nearly zero-bathroom visits in the previous days, they were found in mid-November 2017 and late November 2017.
- The third group consists of four detected timestamps indicating both decreasing and increasing bathroom behavior. Whereas the first two timestamps in the third group indicate decreasing behavior, the last two indicate increasing behavior.
- The last group of detected timestamps consists of six timestamps. The first indicates the beginning of a sudden increase in activity behavior, and the remaining five indicate decreasing activity.

### 5.2. Heatmap representation

We have plotted a heatmap for each subject with the vertical axis as the number of days within a given month and the horizontal axis as the month itself to visualize the normalized bathroom activity for each subject individually to help decision-making by physicians or medical staff.

**Figure 10:**
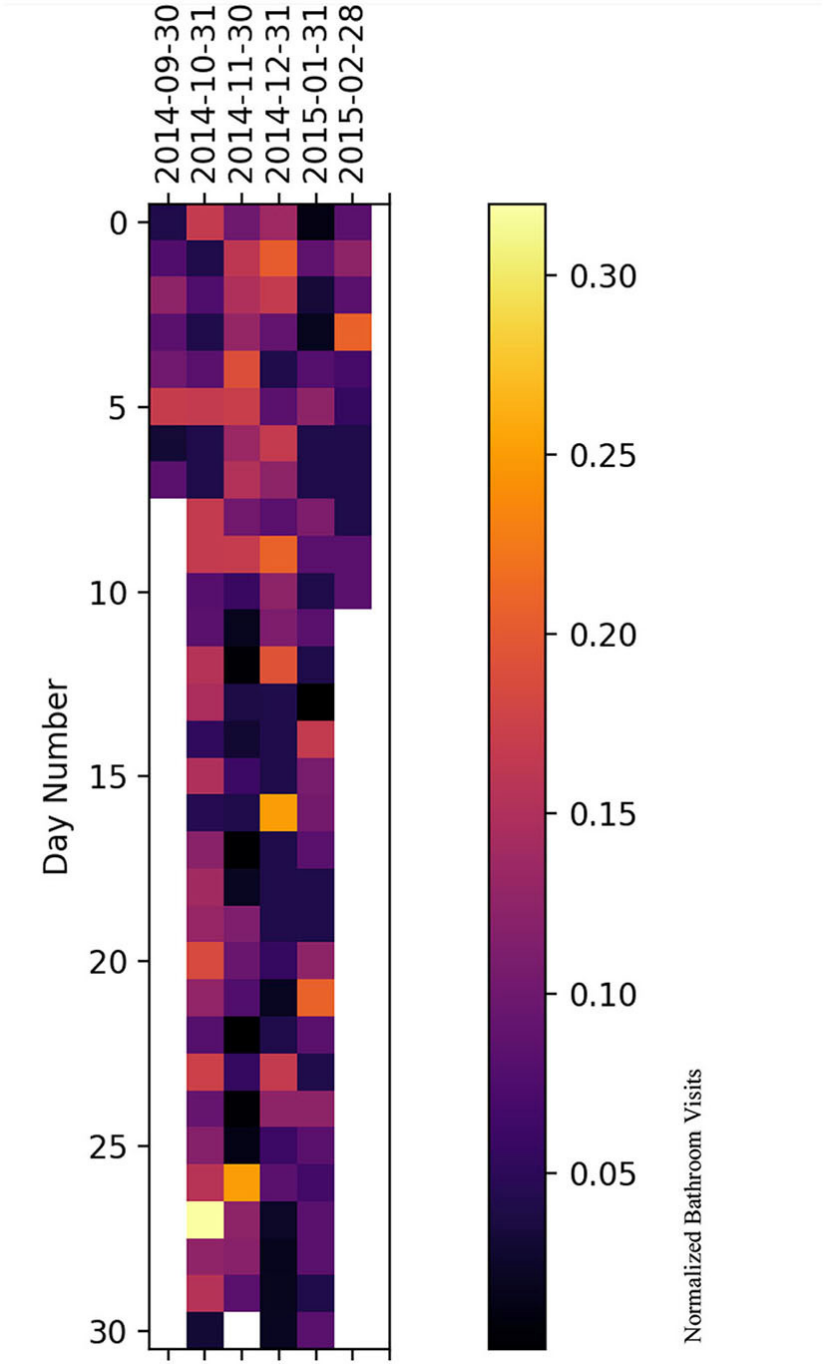
Bathroom activity heatmap for subject A grouped by monthly periods.

For subject A (Figure 10), it is clear that the subject had increased bathroom activity on 1st of November and the 11th of December.

**Figure 11:**
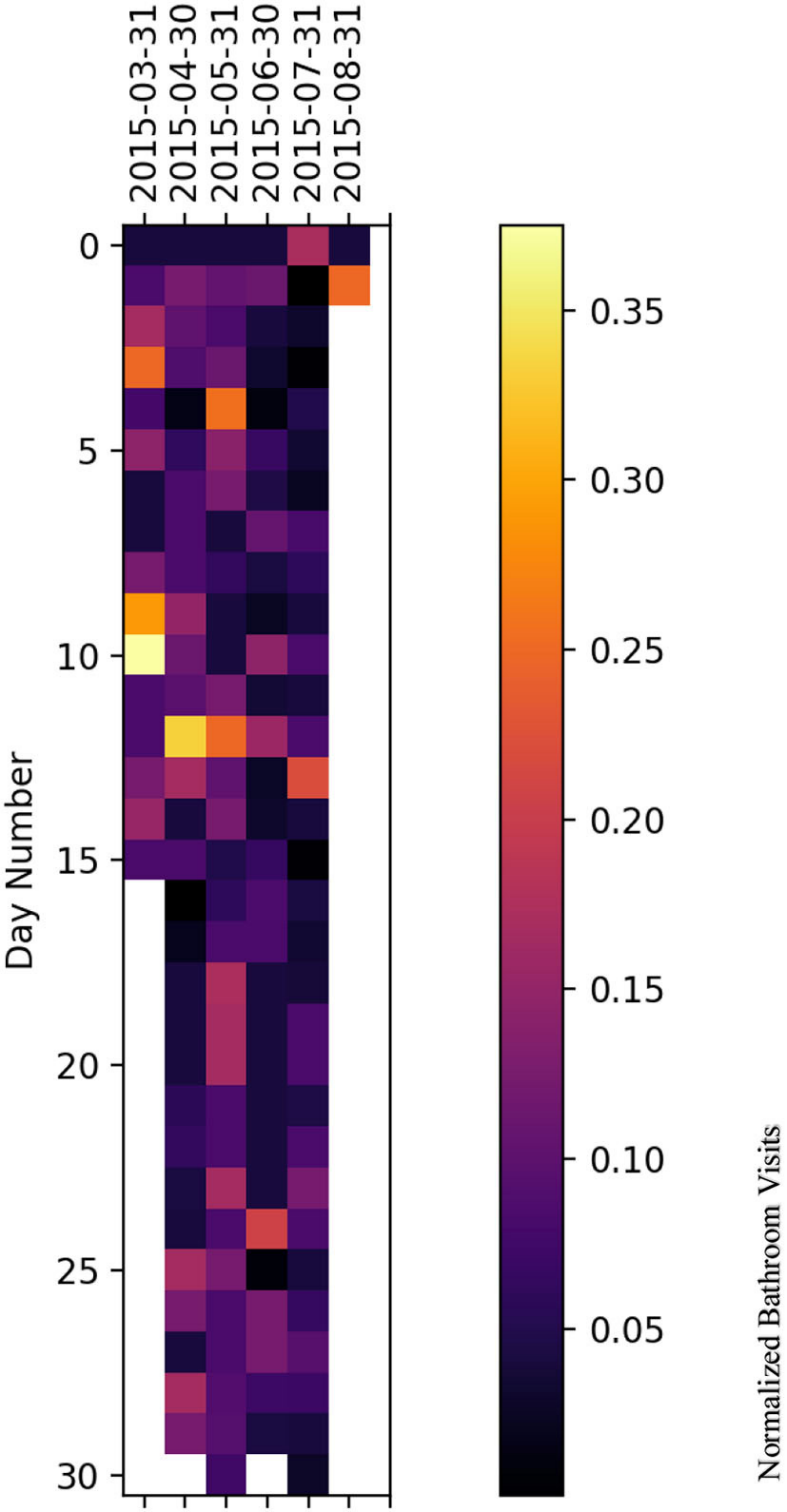
Bathroom activity heatmap for subject B grouped by monthly periods.

For subject B (Figure 11), the subject experienced increased bathroom activity on 8 March 2015 compared to the remaining monitoring days.

**Figure 12:**
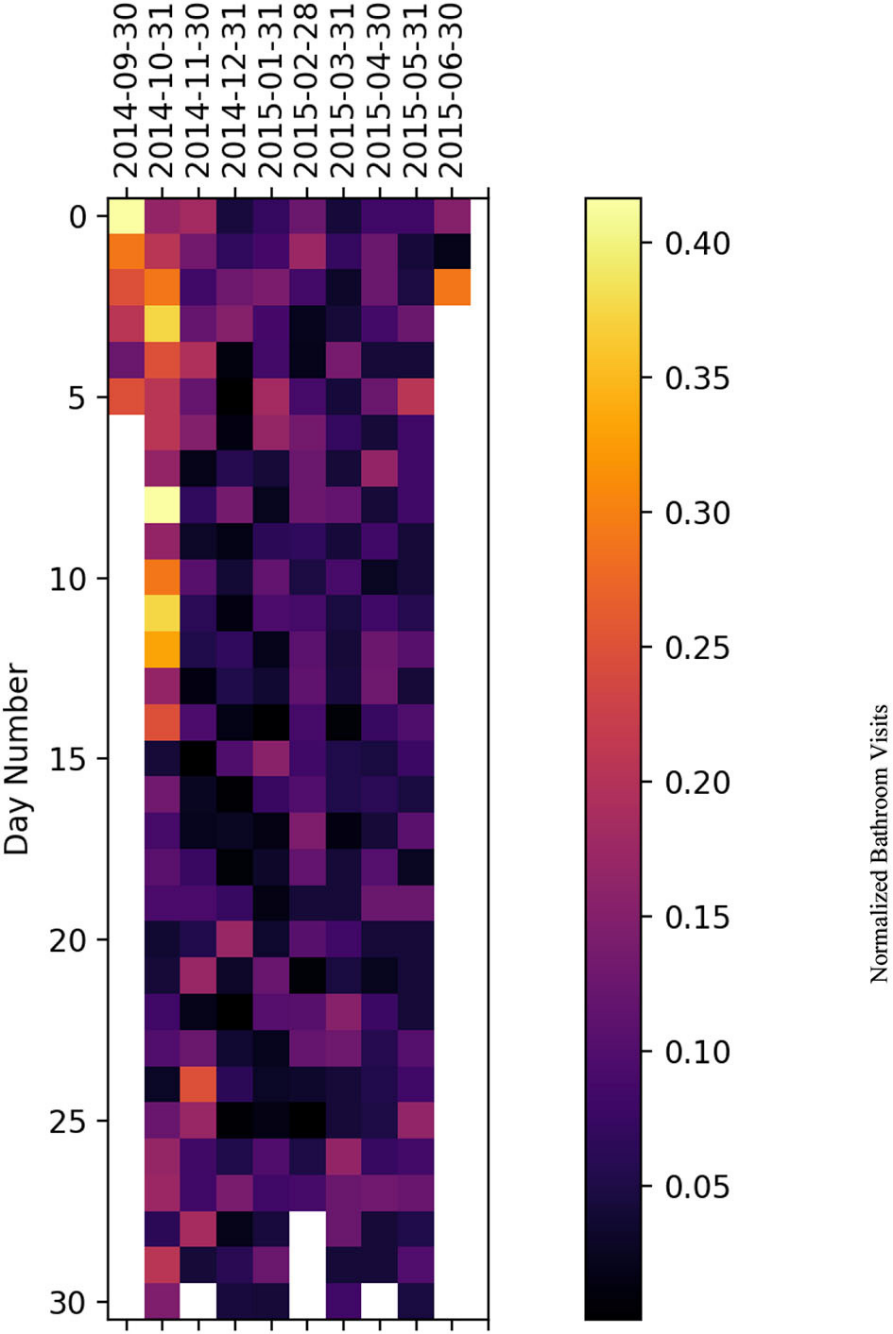
Bathroom activity heatmap for subject C grouped by monthly periods.

For subject C (Figure 12), the subject experienced an increased activity on November 18 and 29 November 2016. Furthermore, there was increased bathroom activity on 28 June 2017, 7 October 2017, 10, 19, and 26 November 2017, and 11 and 13 January 2018.

**Figure 13:**
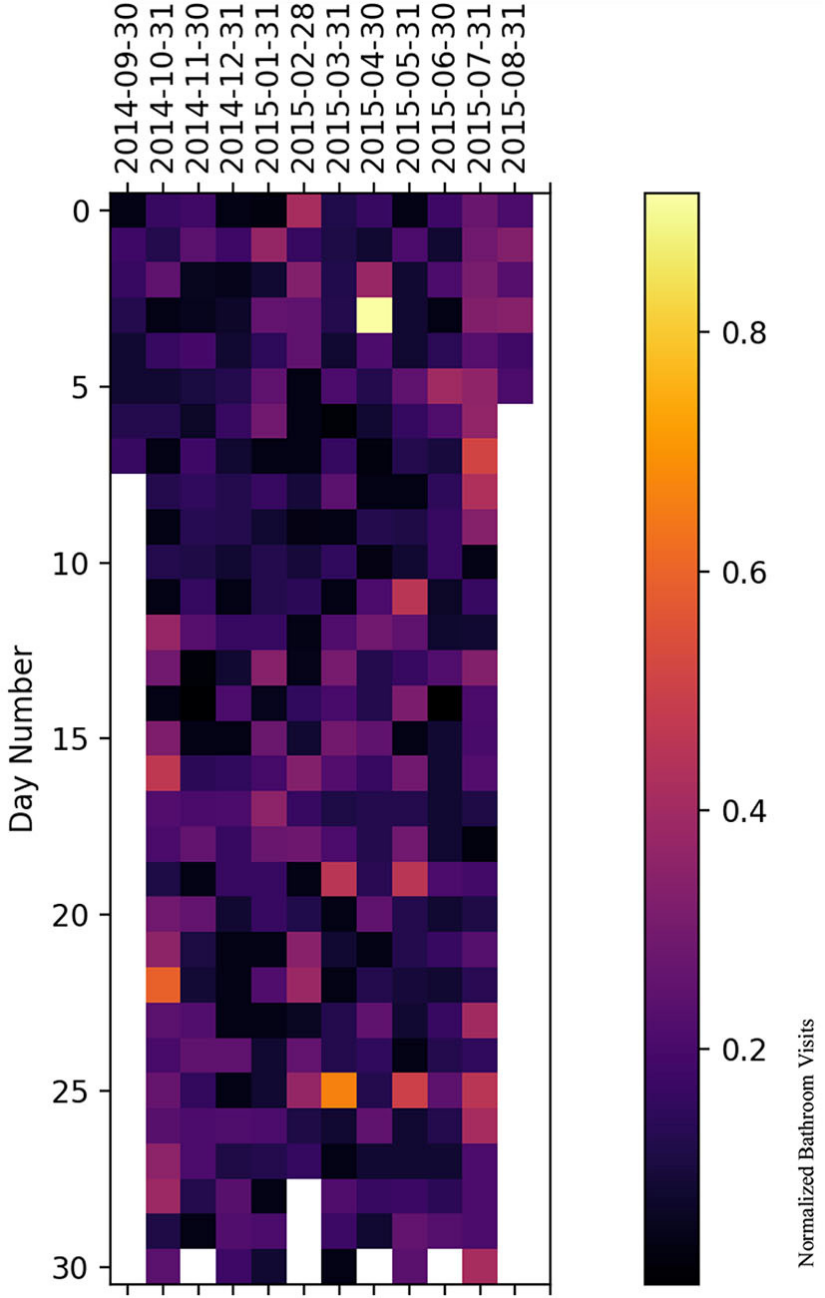
Bathroom activity heatmap for subject D grouped by monthly periods.

For subject D (Figure 13), the subject experienced increased bathroom activity on 12 March 2015 and 3 April 2015.

**Figure 14:**
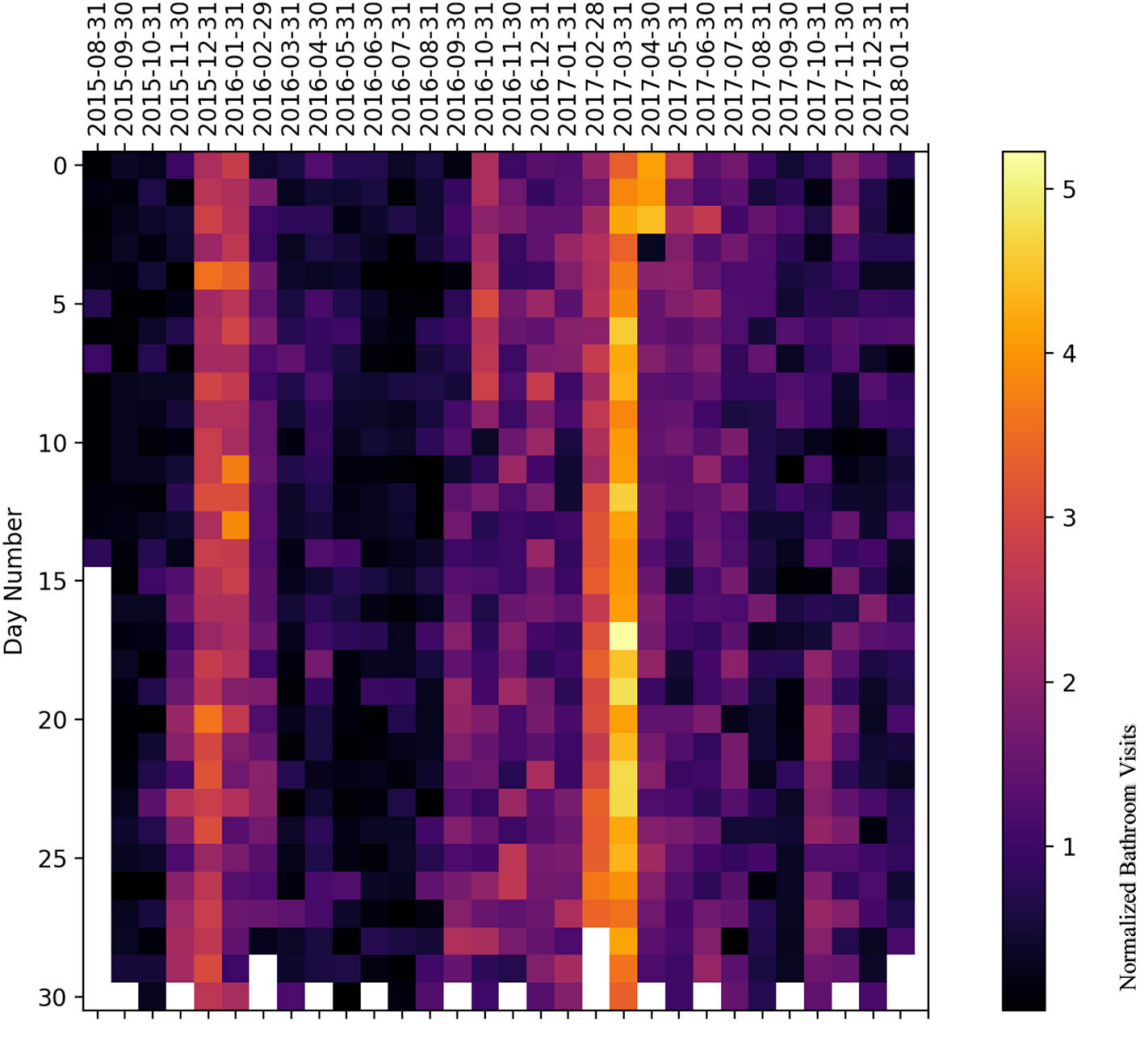
Bathroom activity heatmap for subject E grouped by monthly periods.

For subject E (Figure 14), the subject's behavior shows a decreasing bathroom activity behavior during the period extending from August 2015 to October 2015, and during the period extending from February 2016 to May 2016. The subject experienced increased bathroom behavior during the periods extending from October 2016 to January 2017, from April 2017 to July 2017, and in October 2017. Specifically, increased behaviors were found on 26 and 27 November 2016, 8 December 2016, 1 May 2017, and 3 June 2017.

**Figure 15:**
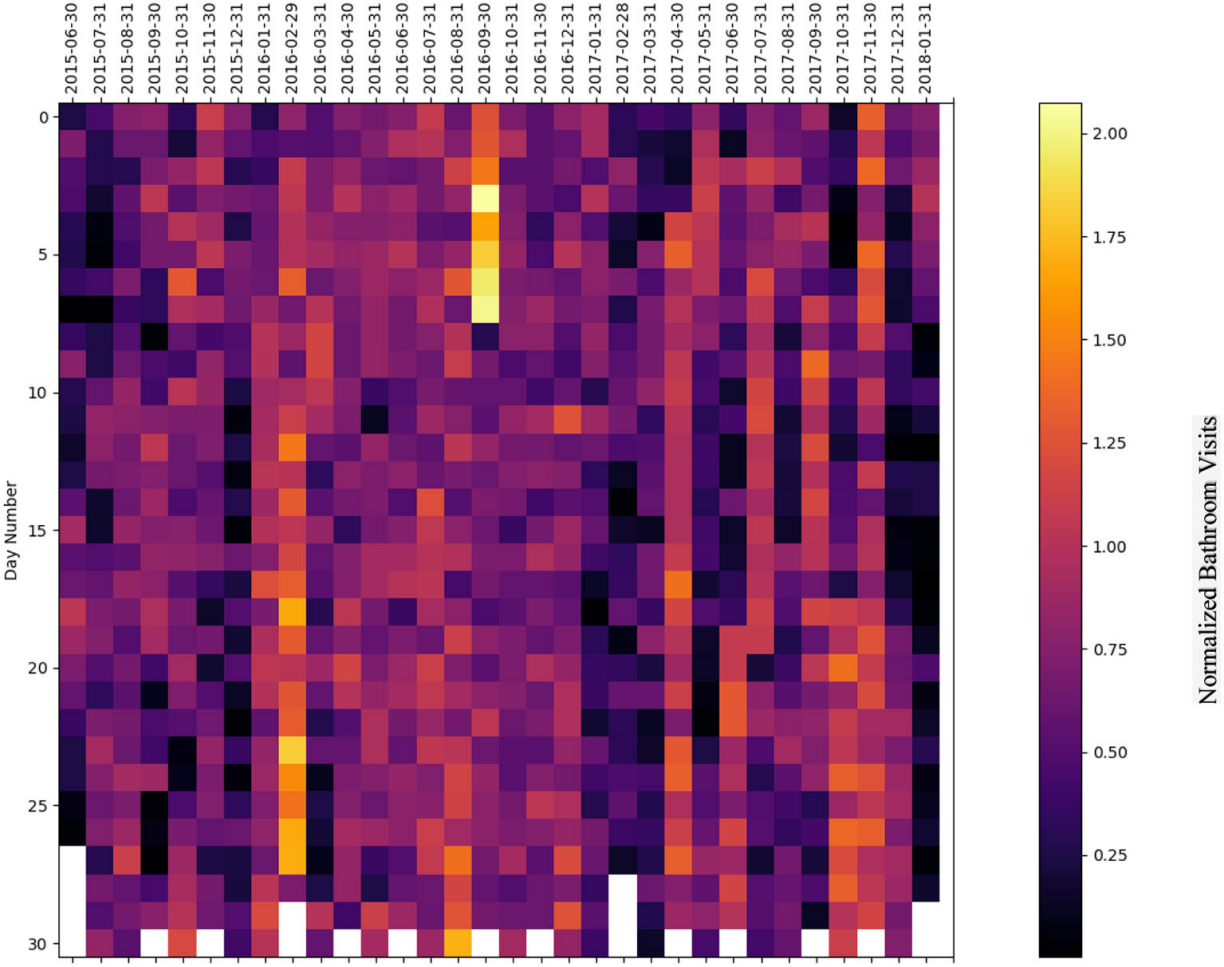
Bathroom activity heatmap for subject F grouped by monthly periods.

For subject F (Figure 15), the subject experienced an increase in bathroom activity in the period extending from October 2016 to January 2017, specifically on 18 October 2016, on 19, 22, and 25 December 2016, and on 13 January 2017.

For subject G (Supplementary Figure 10), the subject experienced an increase in bathroom activity during March 2015, April 2015, March 2016, April 2016, and May 2016. The highest bathroom activity can be found on 2 March 2016, 9 and 19 April 2016, and 4, 5, and 21 May 2016, respectively.

For subject H (Supplementary Figure 11), the subject experienced an increased bathroom activity behavior during September 2014 and October 2014, where the specific days of such an increase were on September 1st 2014 and October 8th 2014. There was a significant decrease in the subject bathroom activity in January 2015, March 2015, April 2015, and May 2015 as well.

For subject I (Supplementary Figure 12), the subject's bathroom activity was in general high. Whereas the highest values of bathroom activity were found in April 2017, June 2017, October 2017 and November 2017.

For subject J (Supplementary Figure 13), the subject's bathroom activity behavior was generally low except for 9 April 2015 when the subject experienced a sudden increase in his bathroom activity behavior.

For subject K (Supplementary Figure 14), the subject, in general, was experiencing an increase in his bathroom activity behavior. However, there were a few days where the bathroom activity was higher, specifically on 15 and 29 November 2016 and on 19 and 22 December 2016.

For subject L (Supplementary Figure 15), the subject, in general, was experiencing an increase in his bathroom activity behavior, where the highest activity value was on 24 April 2017, 4 May 2017, and 14 January 2018.

For subject M (Supplementary Figure 16), the subject was experiencing low bathroom activity, in general, except for October 2016. Typically, the subject experiences the highest activity on 6–8 October 2016.

For subject N (Supplementary Figure 17), the subject was experiencing high bathroom activity in July 2017, particularly on 1, 2, 3, 16, and 19 July 2017.

For subject P (Supplementary Figure 18), the subject was experiencing high bathroom activity on 1 April 2017 and 10 June 2017.

For subject V (Supplementary Figure 19), the general bathroom activity behavior of the subject was low except for 3 January 2018 where the subject had the highest bathroom activity value.

### 5.3. Insight

We implemented our system to help collaborating medical staff (doctors, caregivers, and nursing) better follow the subjects (e.g., to notify the medical staff of possible excretory function disorders experienced by the subject under investigation). To this end, we have compared our detection results with those obtained by medical staff.

According to medical staff reports, two subjects had excretory function disorders, subject I was reported to have a UTI, and subject H was reported to have IBS-related symptoms such as vomiting, diarrhea, and spending longer in the bathroom.

- For subject F, three consecutive increases affect the daily evolution of toilet entries. Subject F entered more frequently the toilet on 2015-09, 2015-10-10, 2015-10-20, 2015-10, 2016-03, 2017-04, 2017-06, 2017-10, and 2017-11. Collaborating medical staff confirmed urinary infection symptoms starting from 2015-10. They observed frequent toilet entries, vomiting, and diarrhea until 2016-04.
  ∘ Our system detected increased toilet visits, suggesting urinary infection events a few weeks before they were observed by the medical staff. The change detection results obtained by our approach conformed with those obtained by the medical staff, which means we were able to report bathroom activity behavior change that helped medical staff intervene early to solve the situation.
  ∘ In addition, the normalized bathroom activity value that complied with the medical staff report was equivalent to 1.2 bathroom visits, meaning that it sums up to approximately 28 bathroom visits per day.
- For subject H, we found one significant decrease that affected the daily evolution of toilet entries; this decrease occurred on 2015-01-01. The nursing home team observed an increase in mobility impairments for subject H in 2015-01-15. By this time, subject H was no longer able to go independently to the toilet, as fecal elimination was observed both on the bed and all over the room.
  ∘ The medical staff report matched our detection results as to where we were able to report such degradation of bathroom activity behavior.

As for the other subjects, there were no reported issues/disorders by the medical staff. However, our analysis confirmed changes for all subjects. These changes were more notable for subject J on 2015-04-09, subject G in late April 2015, in March, April, and May 2016, subject F starting from October 2016 to January 2017, subject A on 1 November 2014 and 11 December 2014, subject B on 8 March 2015, subject C from October 2016 to January 2018, subject D on 3 April 2015, subject E starting from 25 November 2016 to October 2017, subject K on 15 November 2016 and 19 and 22 December 2016, subject L on 24 April 2017, subject M on October 2016, subject N on July 2017, subject P on 1 April 2017, and subject V on 4 January 2018.

We claim that these detected changes were due to UTI as our proposed algorithm counts the number of bathroom visits per day, or, in other words, accounts for the bathroom visit frequency, which is related to UTI. These changes may be related to situations that can result in more serious problems in future, which is why medical doctors need to be made aware of these behavior changes as soon as they are detected.

## 6. Conclusion

The Internet of Things (IoT) and artificial intelligence (AI)-based monitoring technology provide new objective information on daily living activities that complete classical medical observations. In this study, we proposed an attempt to effectively translate medical staff needs by deploying unobtrusive IoT monitoring technologies (e.g., environmental sensors and sensor-enhanced devices) for early detection of possible changes in health status. We focused on excretory functional disorders in older adults associated with urinary tract infections (UTIs) and irritable bowel syndrome (IBS).

Our IoT and AI-based approach consists of two parts: the first part is to calculate the bathroom visit frequency per day from acquired raw motion sensor data, and the second part detects the activity change based on a window-based calculation of the Pruned Exact Linear Time (PELT). With our approach, we were able to detect bathroom activity disorders by monitoring the bathroom activity of subjects on a daily basis across extended periods of time. In addition, we showed that we could help medical staff follow the health status of two subjects (i.e., H and I) based on our bathroom activity change alerting features to better diagnose and treat possible disorders.

In addition, we were able to establish a primary threshold value for bathroom activity. Specifically, subjects having approximately 1.2 normalized bathroom visits per day should be assumed to experience UTI, as confirmed by medical staff reports.

We have also detected changes that we claim could be associated with UTI but that were not noticed by caregivers. These behavior changes may be related to situations that can result in more serious problems for older adults in future. That is why medical doctors need to be aware of these changes as soon as they are detected. So, our system can alert medical staff in response to these unusual situations.

Our implemented algorithm accounts for the difference between the frequency of bathroom visits in the current time window and the past time window. For better identification of IBS, we are working on another algorithm that accounts for the time spent in the bathroom. We have tested that algorithm in the context of detecting different daily indoor activities using a thermal sensor array (TSA) system (46), and we expect to get promising results for both IBS and UTI.

In this study, we have highlighted how IoT and AI technologies can advance medical evaluations and upgrade medical assessments by offering novel and objective insights into everyday activities, where the use of sensor observations assists medical professionals in identifying situations that require more in-depth examination, supporting their medical assessments, and detecting patterns of decline that may not be obvious during standard office appointments.

## Data availability statement

The data analyzed in this study is subject to the following licenses/restrictions: European project. Requests to access these datasets should be directed to HA, hamdi.aloulou@gmail.com.

## Ethics statement

The studies involving human participants were reviewed and approved by Comités de Protection des Personnes (CPP) Ile-de-France VI Pitié-Salpêtrière Hospital Group. The patients/participants provided their written informed consent to participate in this study.

## Author contributions

BA involved in medical concept proposal, conceptualization, data collection, funding acquisition, methodology, review, editing, writing, and supervision. HM involved in medical background formulation, investigation, data curation, methodology, data analysis conceptualization, formal analysis, visualization, and writing and editing. HA involved in medical concept proposal, conceptualization, data collection, data curation, investigation, methodology, writing, reviewing, and participate in writing and editing. MM involved in data acquisition funding, data collection, data curation, and reviewing. FB involved in data curation, methodology, formal analysis, visualization, review, editing, and supervision.
