# Supplementary material for: IoT in medical diagnosis: detecting excretory functional disorders for Older adults via bathroom activity change using unobtrusive IoT technology
Source: Front Public Health. 2023 Sep 29;11:1161943. doi: 10.3389/fpubh.2023.1161943 (PMC10574436; doi:10.3389/fpubh.2023.1161943)
Supplement: Supplementary file 1 [file Presentation_1.zip › 1161943_Supplementary_Material.docx]

**Supplementary Materials**


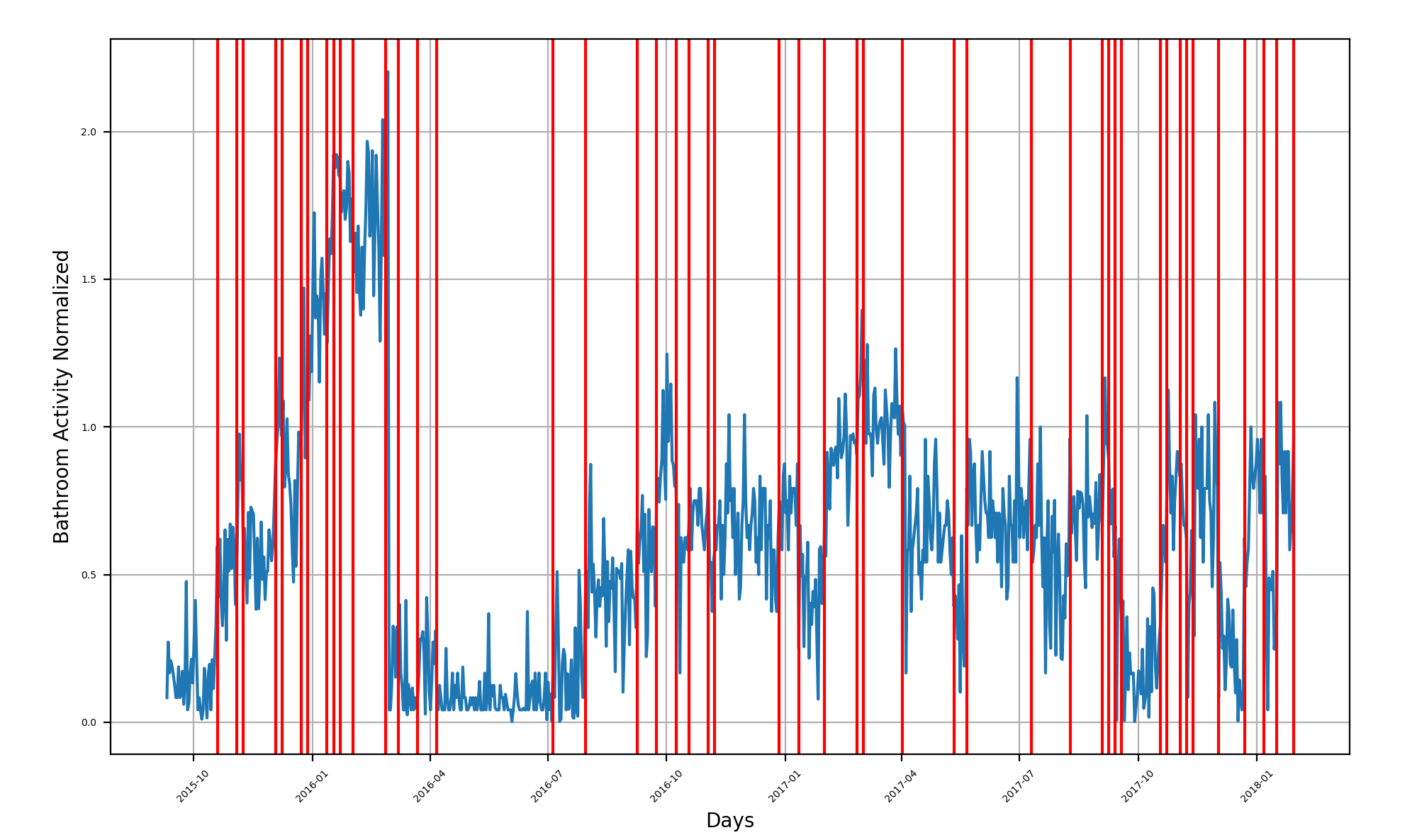


Figure 1. Bathroom activity time series for subject H, with detected change time stamps overlapped as vertical lines.


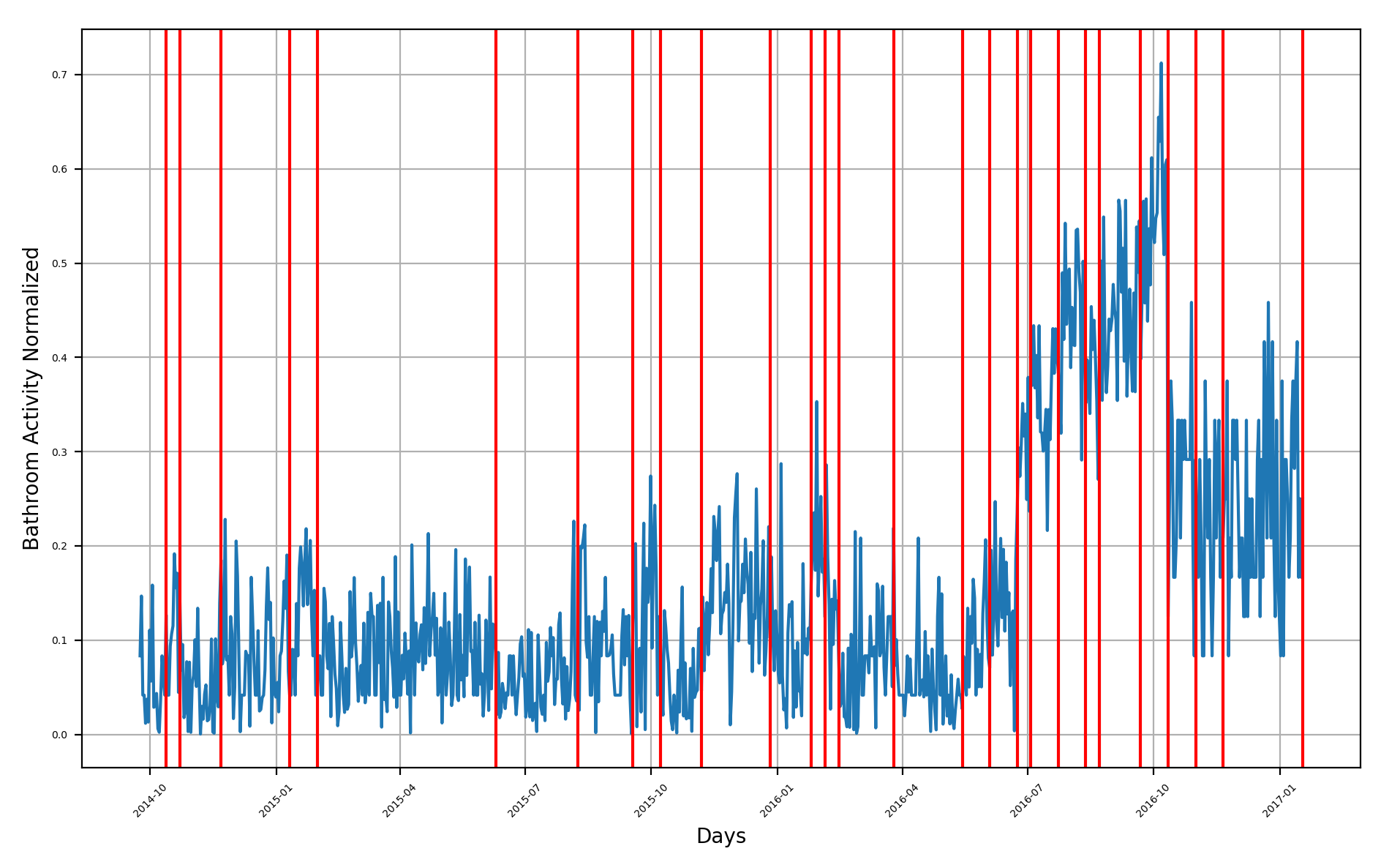


Figure 2. Bathroom activity time series for subject I, with detected change time stamps overlapped as vertical lines.


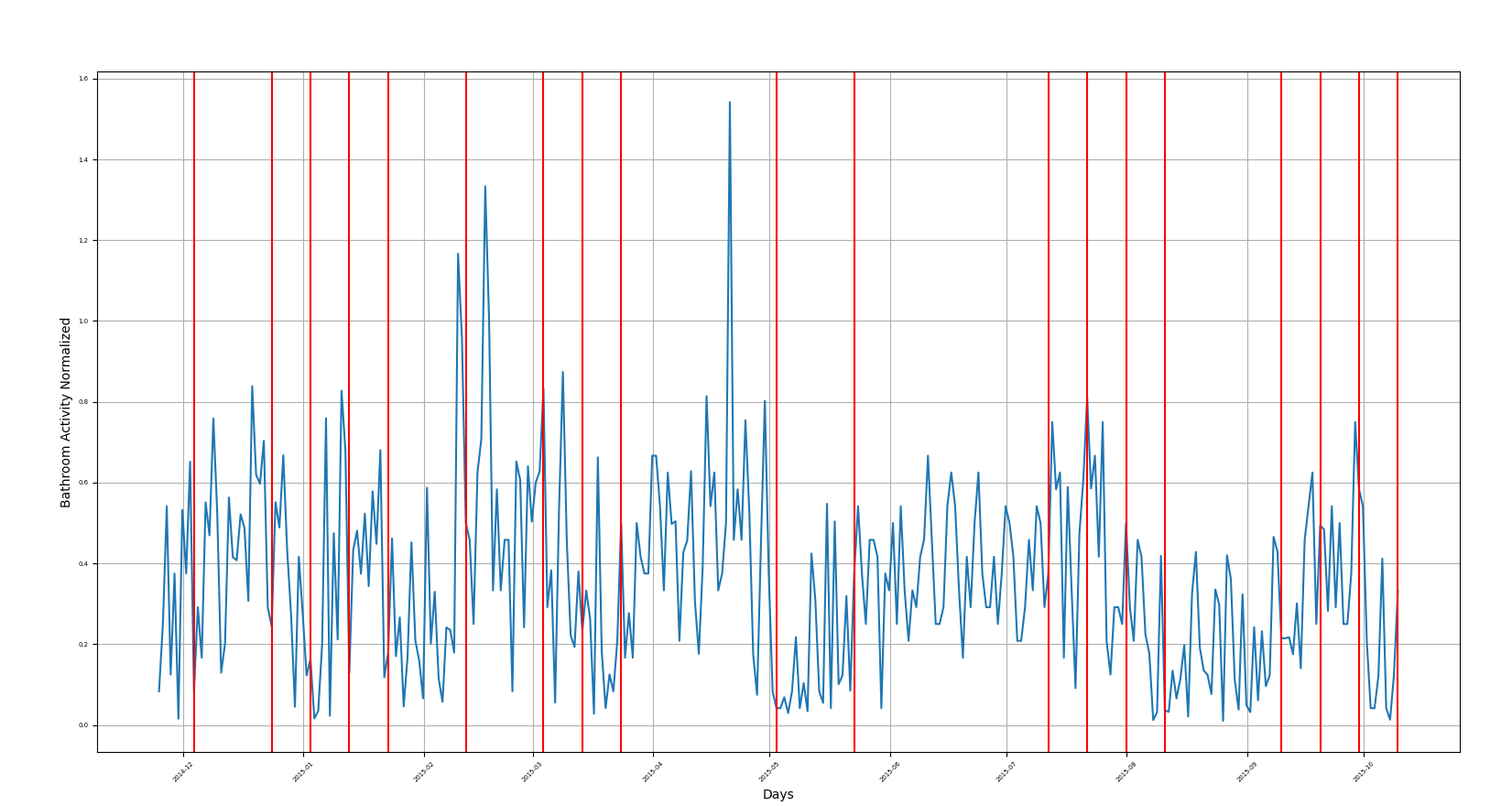


Figure 3. Bathroom activity time series for subject J, with detected change time stamps overlapped as vertical lines.


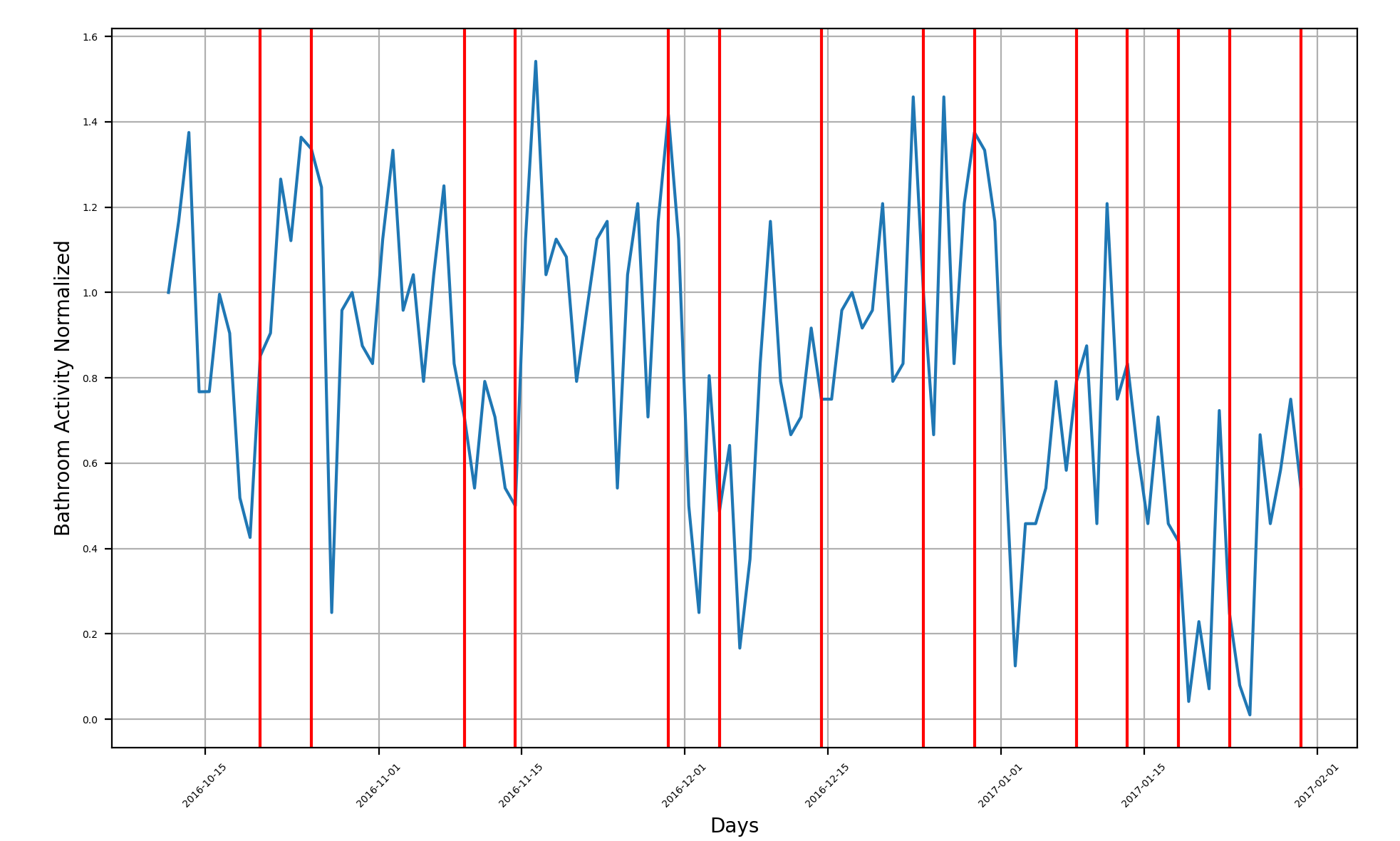


Figure 4. Bathroom activity time series for subject K, with detected change time stamps overlapped as vertical lines.


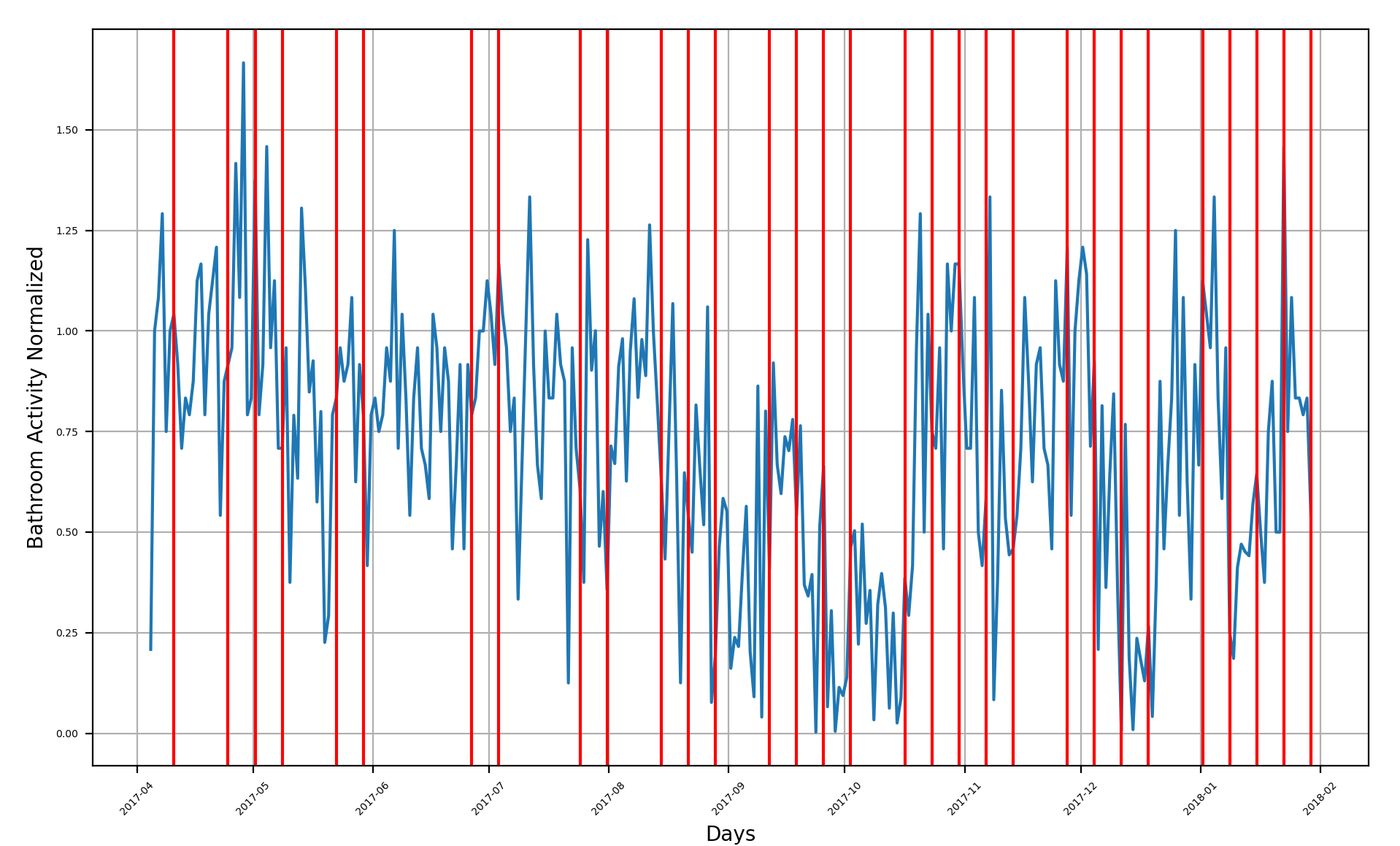


Figure 5. Bathroom activity time series for subject L, with detected change time stamps overlapped as vertical lines.


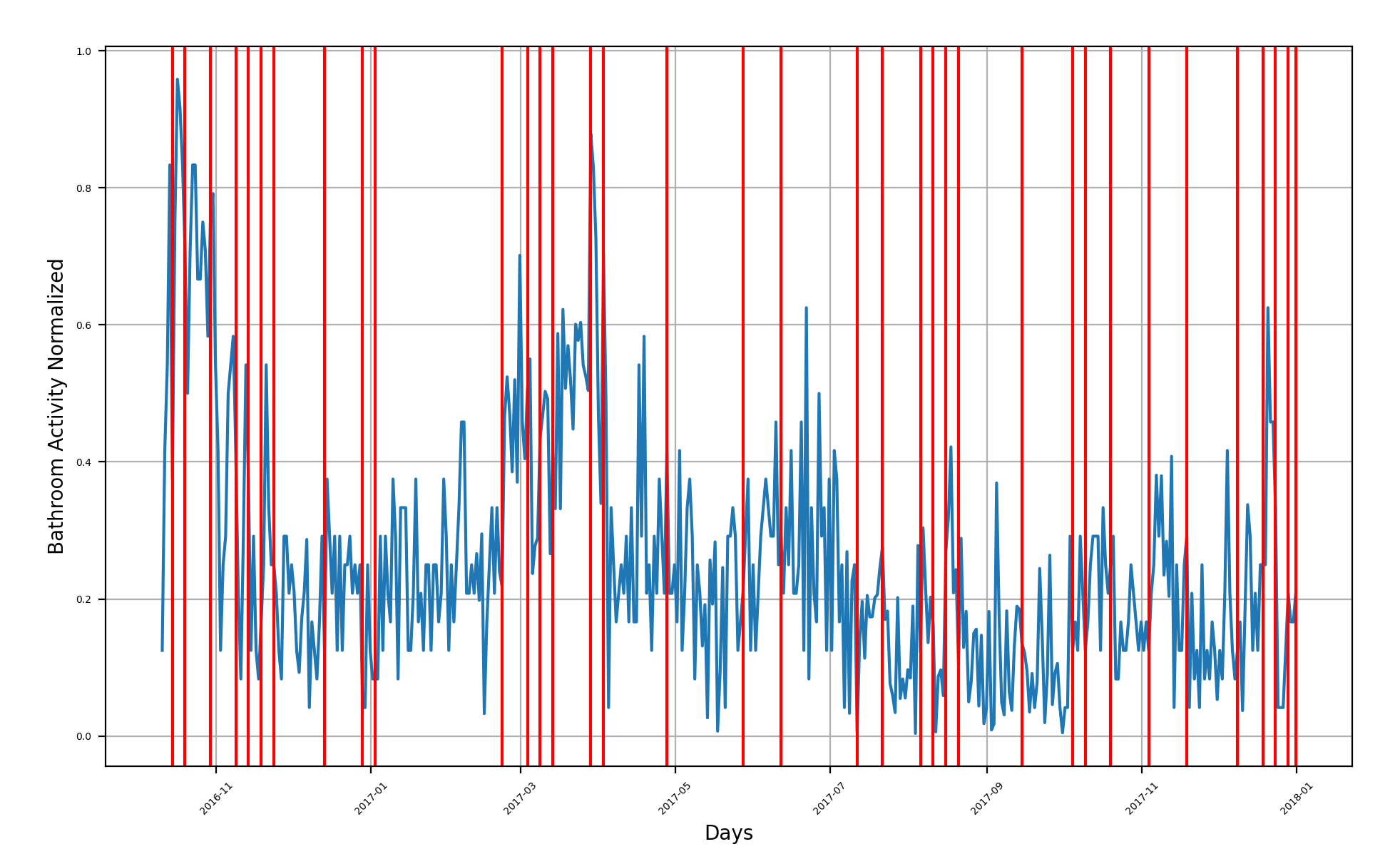


Figure 6. Bathroom activity time series for subject M, with detected change time stamps overlapped as vertical lines.


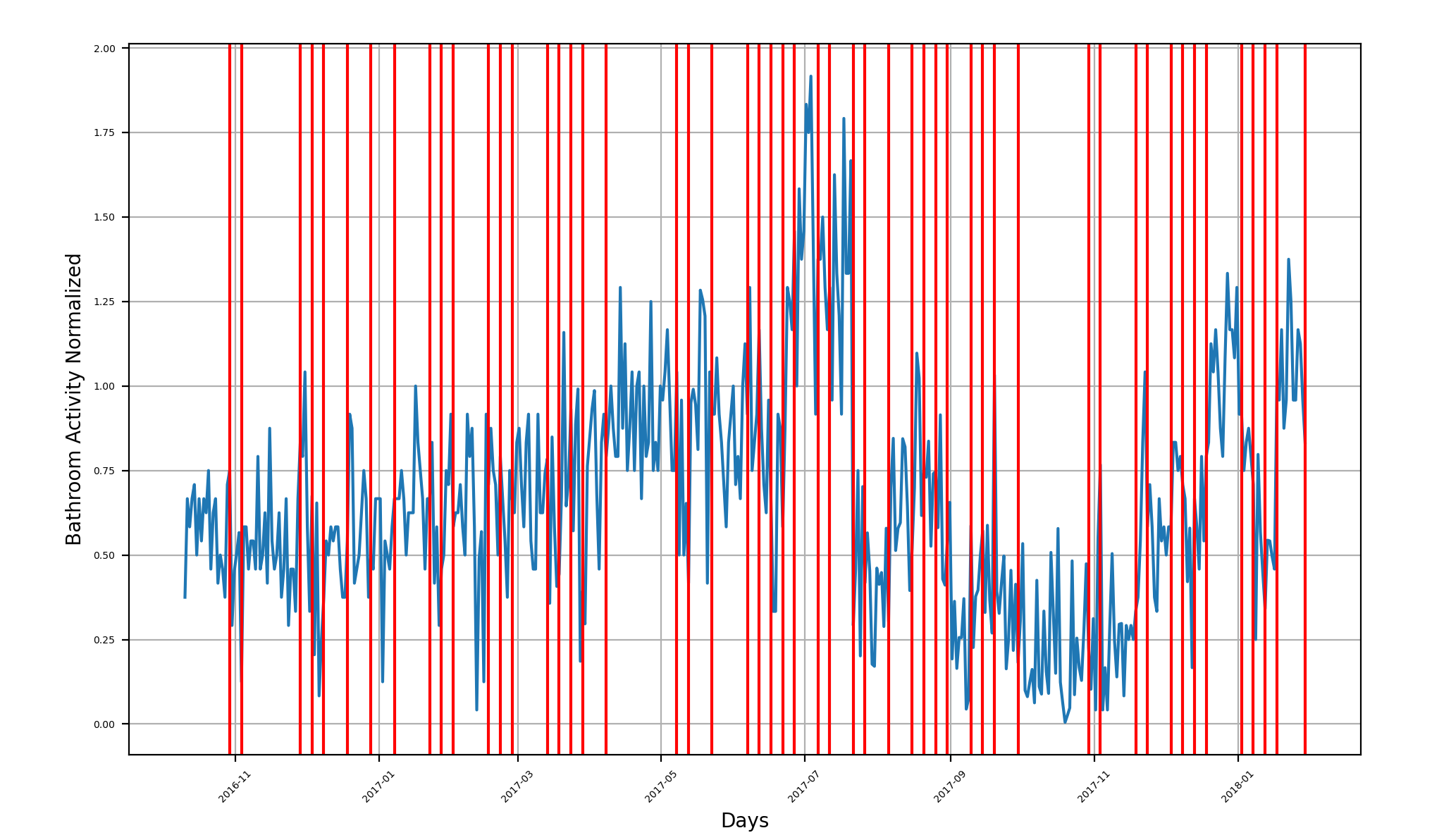


Figure 7. Bathroom activity time series for subject N, with detected change time stamps overlapped as vertical lines.


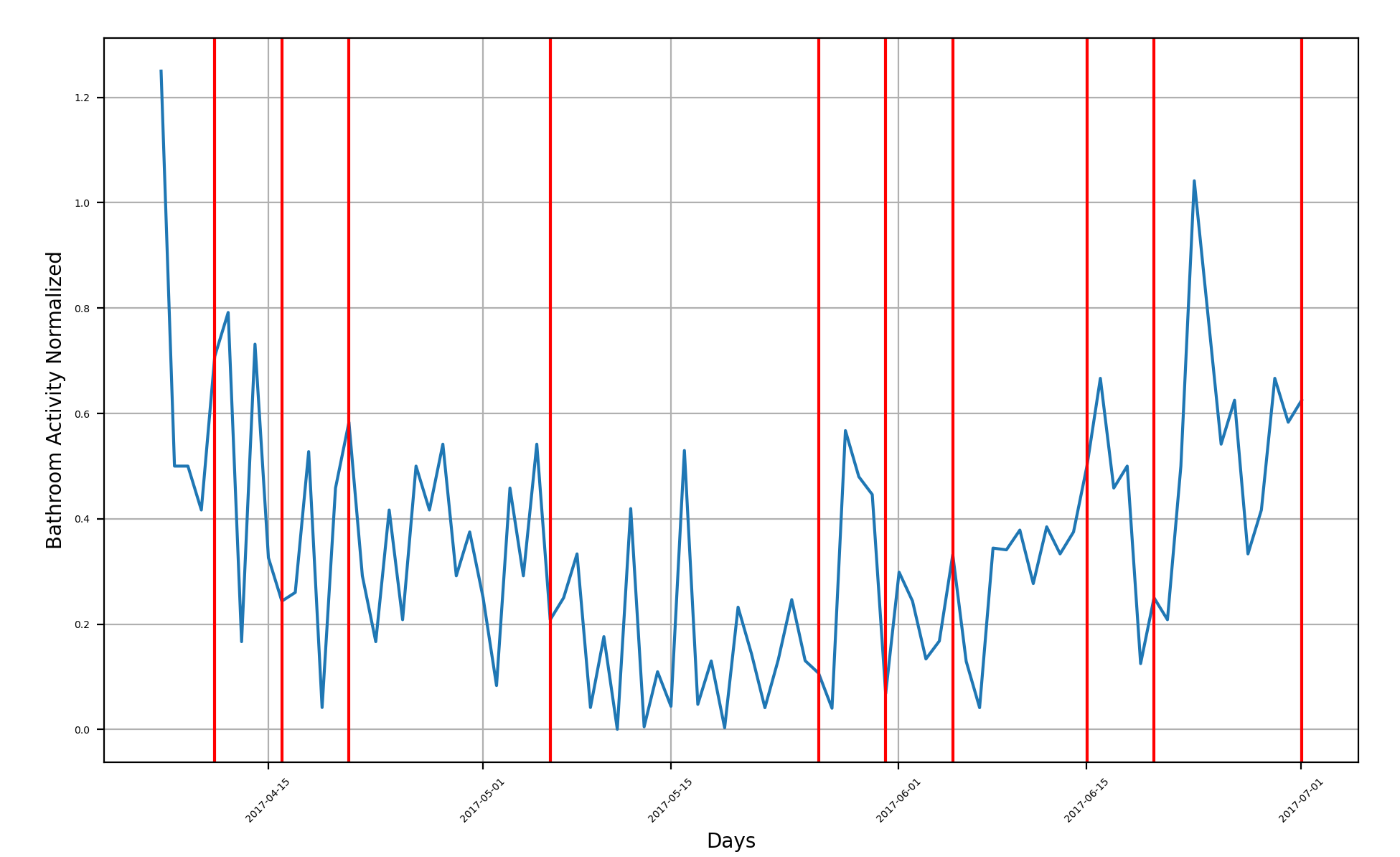


Figure 8. Bathroom activity time series for subject P, with detected change time stamps overlapped as vertical lines.


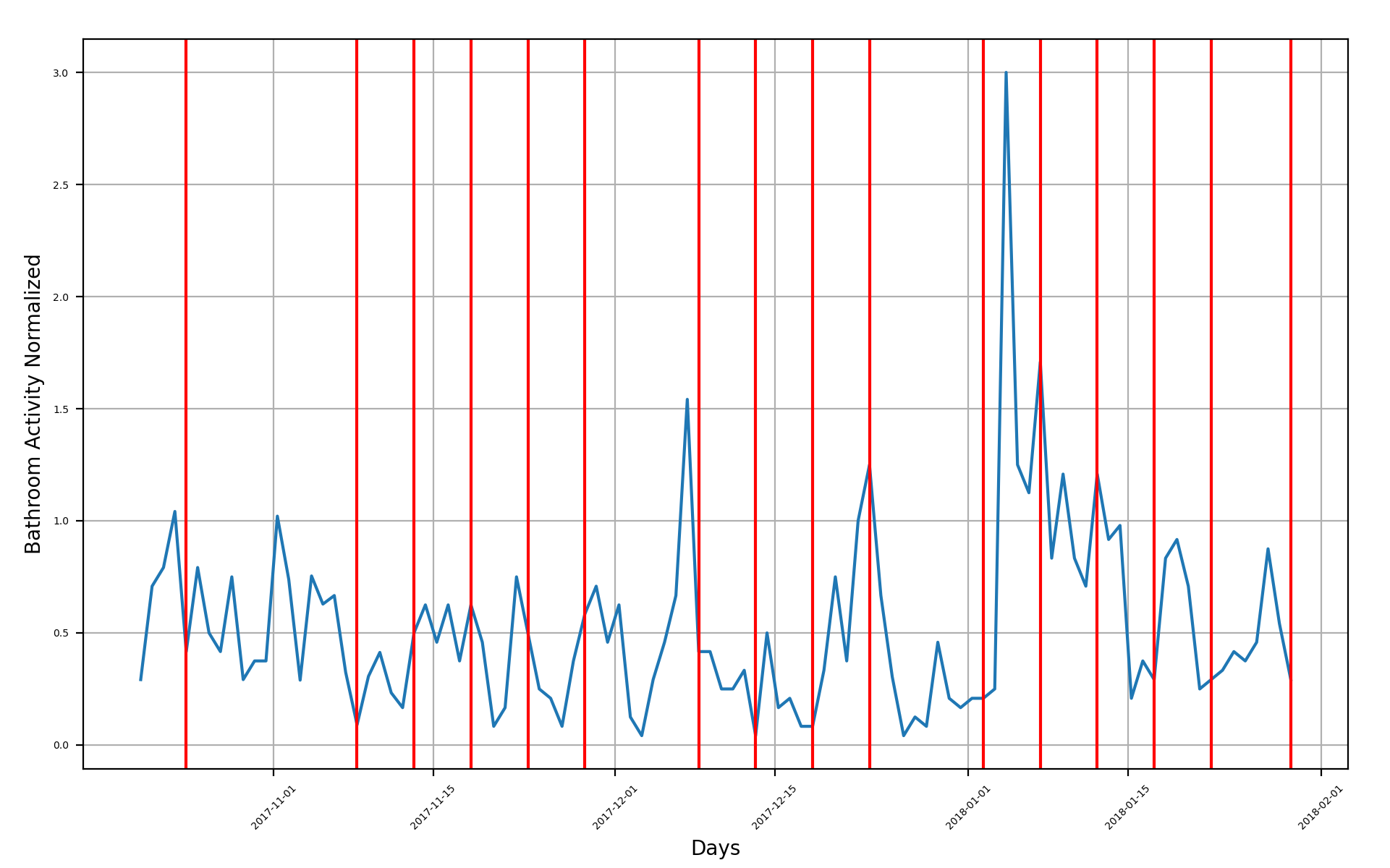


Figure 9. Bathroom activity time series for subject V, with detected change time stamps overlapped as vertical lines.


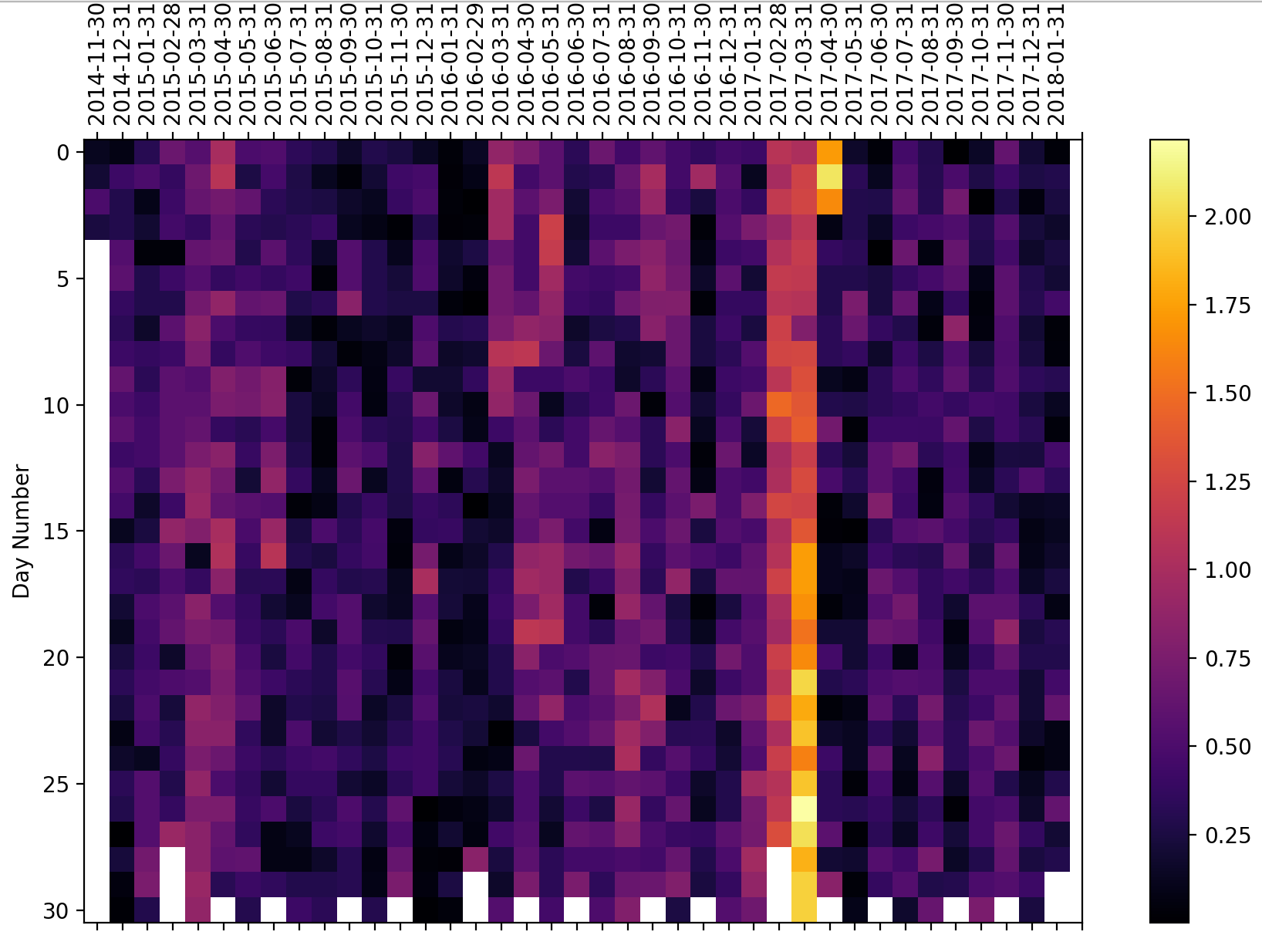


Normalized Bathroom Visits

Figure 10. Bathroom activity heatmap for subject G grouped by monthly periods.


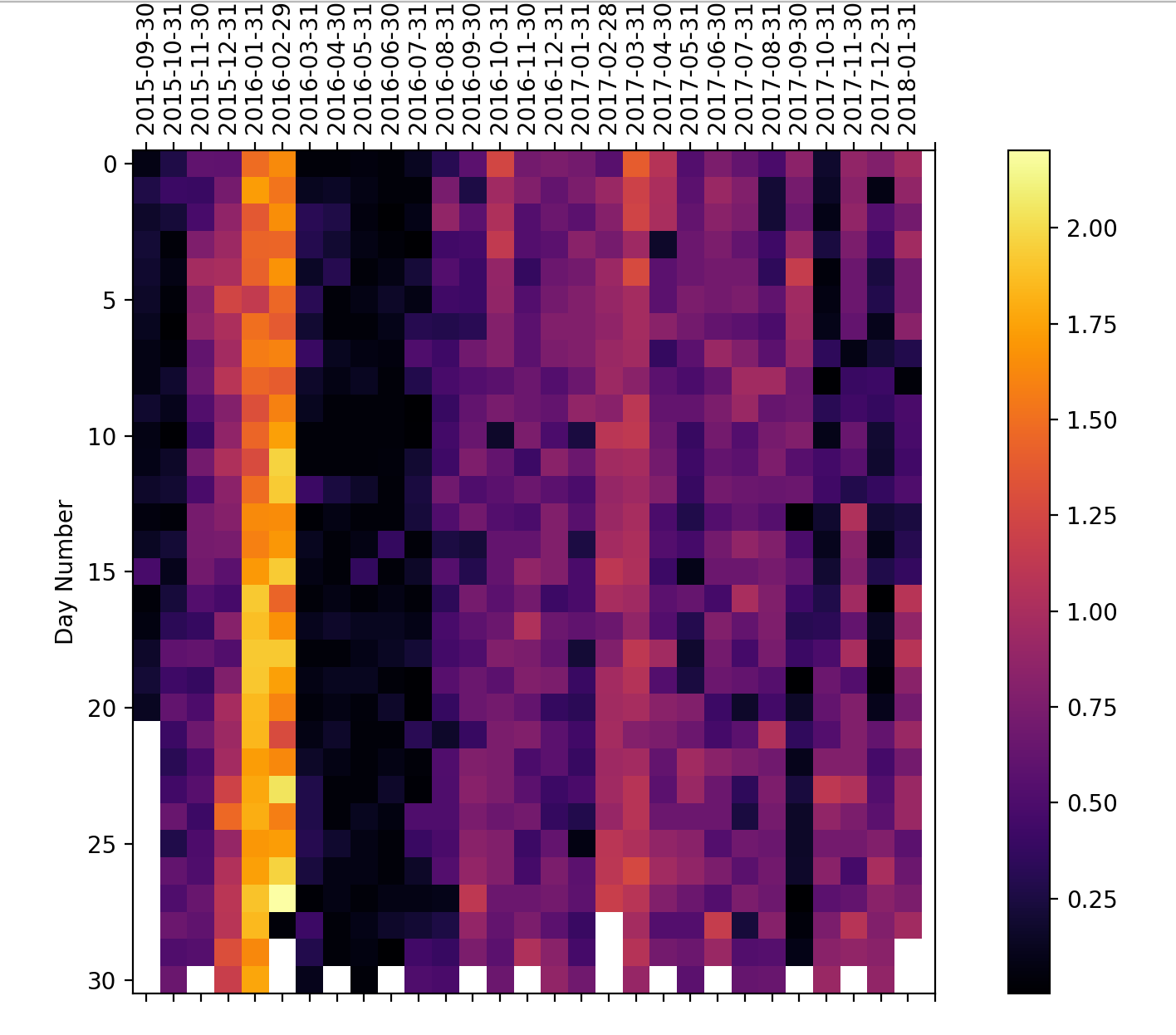


Normalized Bathroom Visits

Figure 11. Bathroom activity heatmap for subject H grouped by monthly periods.


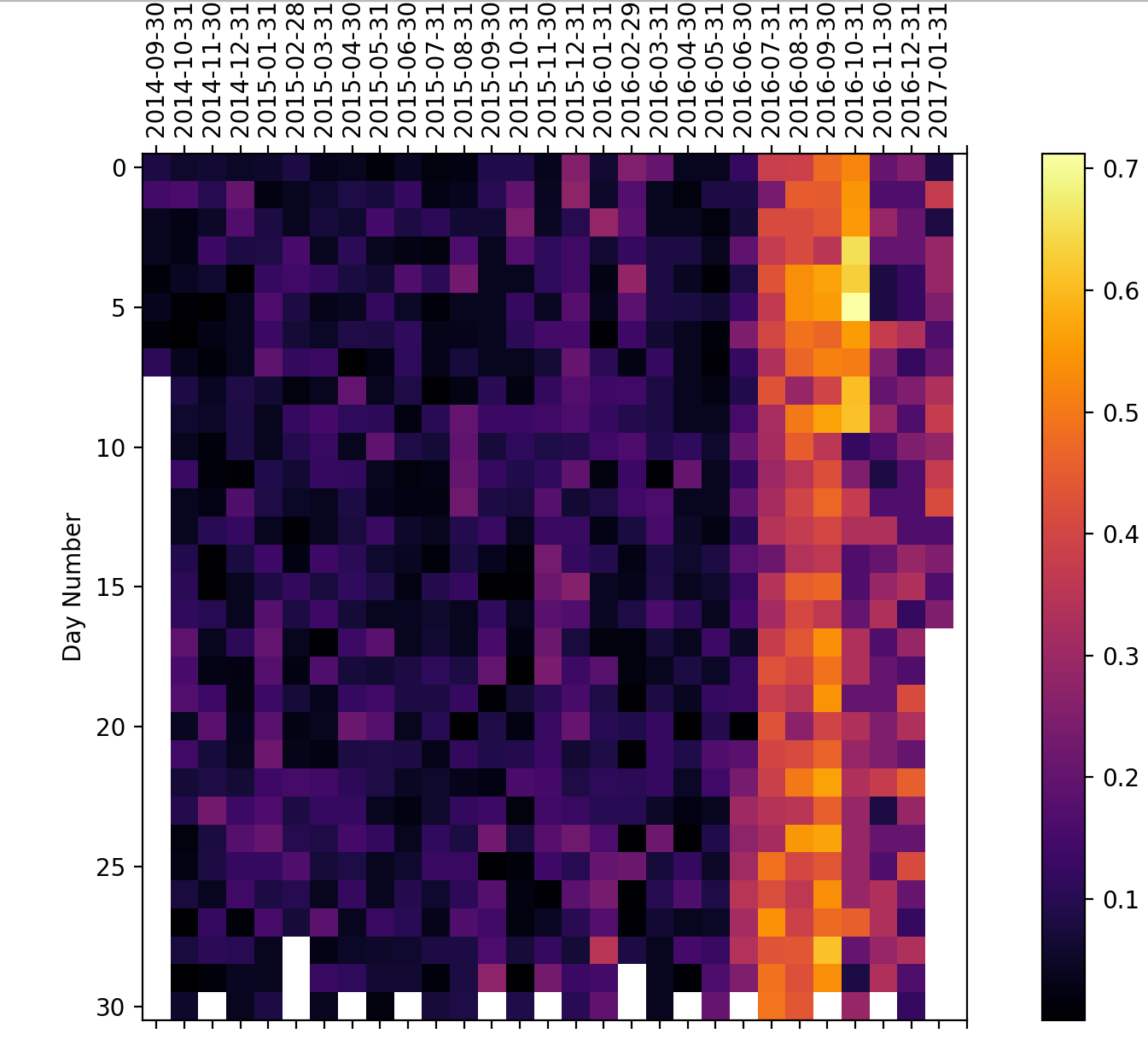


Normalized Bathroom Visits

Figure 12. Bathroom activity heatmap for subject I grouped by monthly periods.


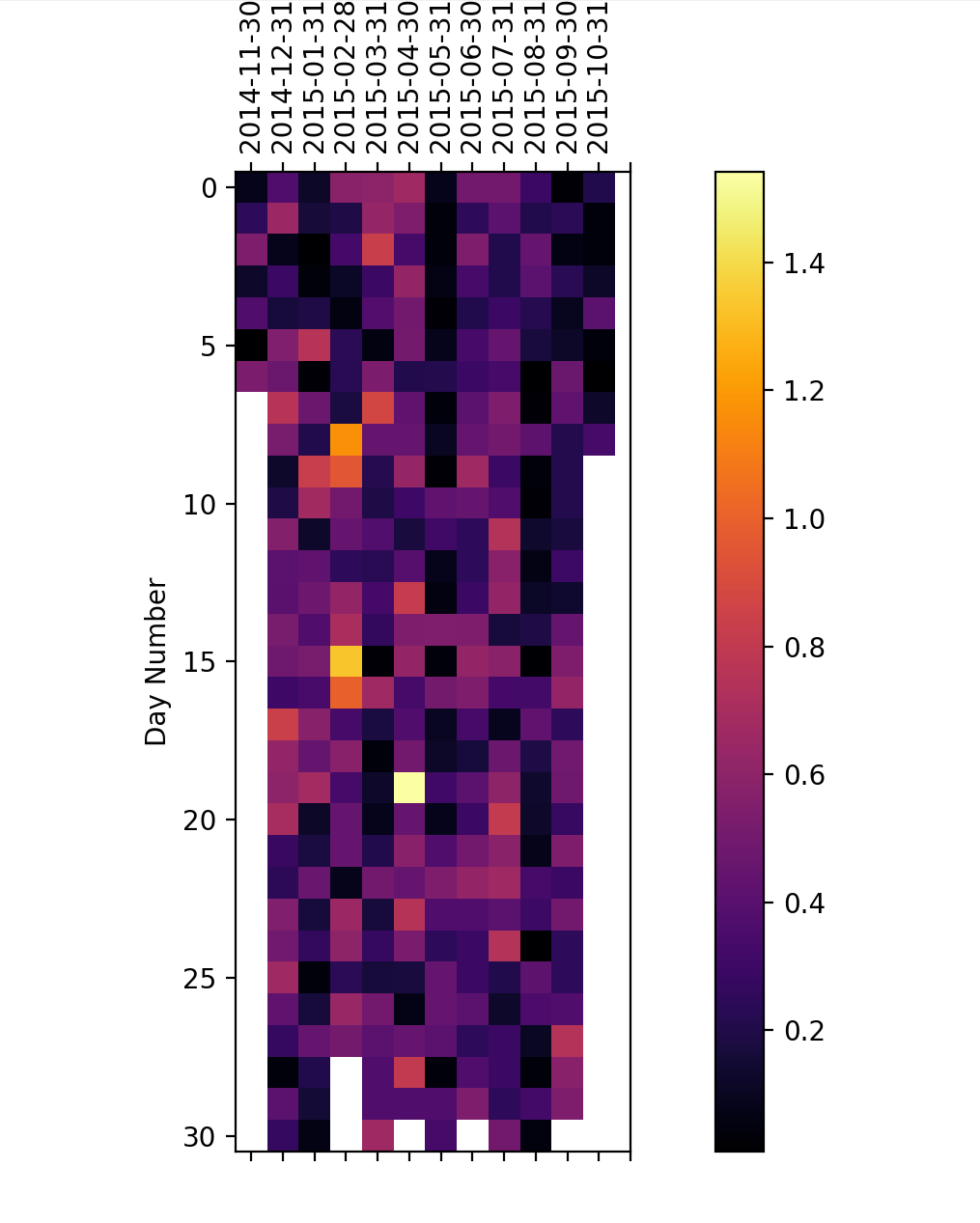


Normalized Bathroom Visits

Figure 13. Bathroom activity heatmap for subject J grouped by monthly periods.


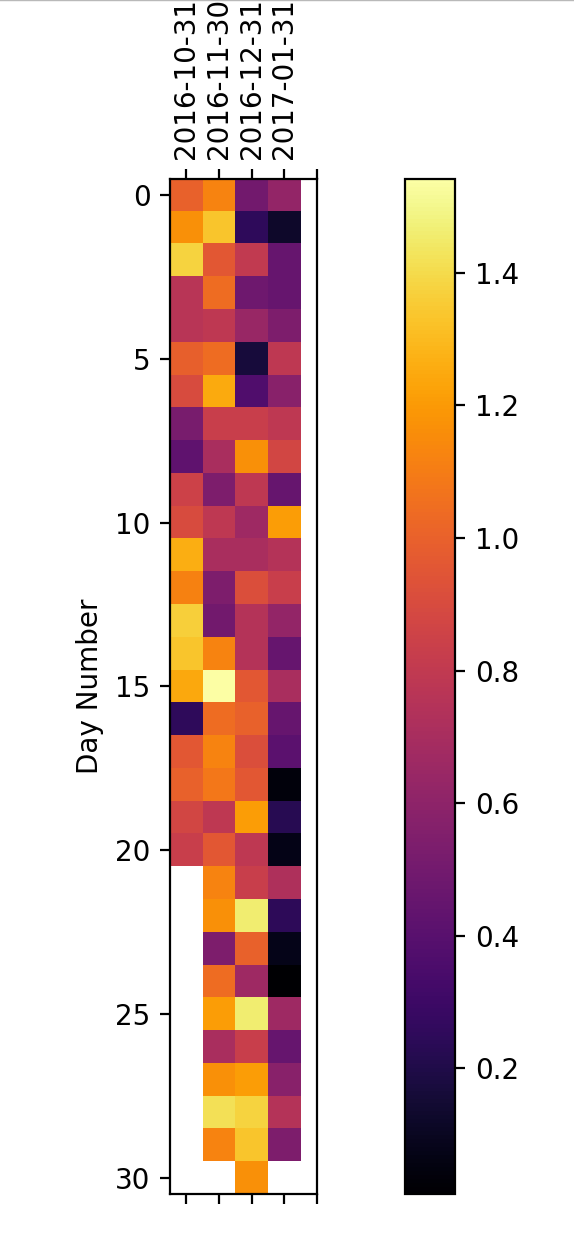


Normalized Bathroom Visits

Figure 14. Bathroom activity heatmap for subject K grouped by monthly periods.


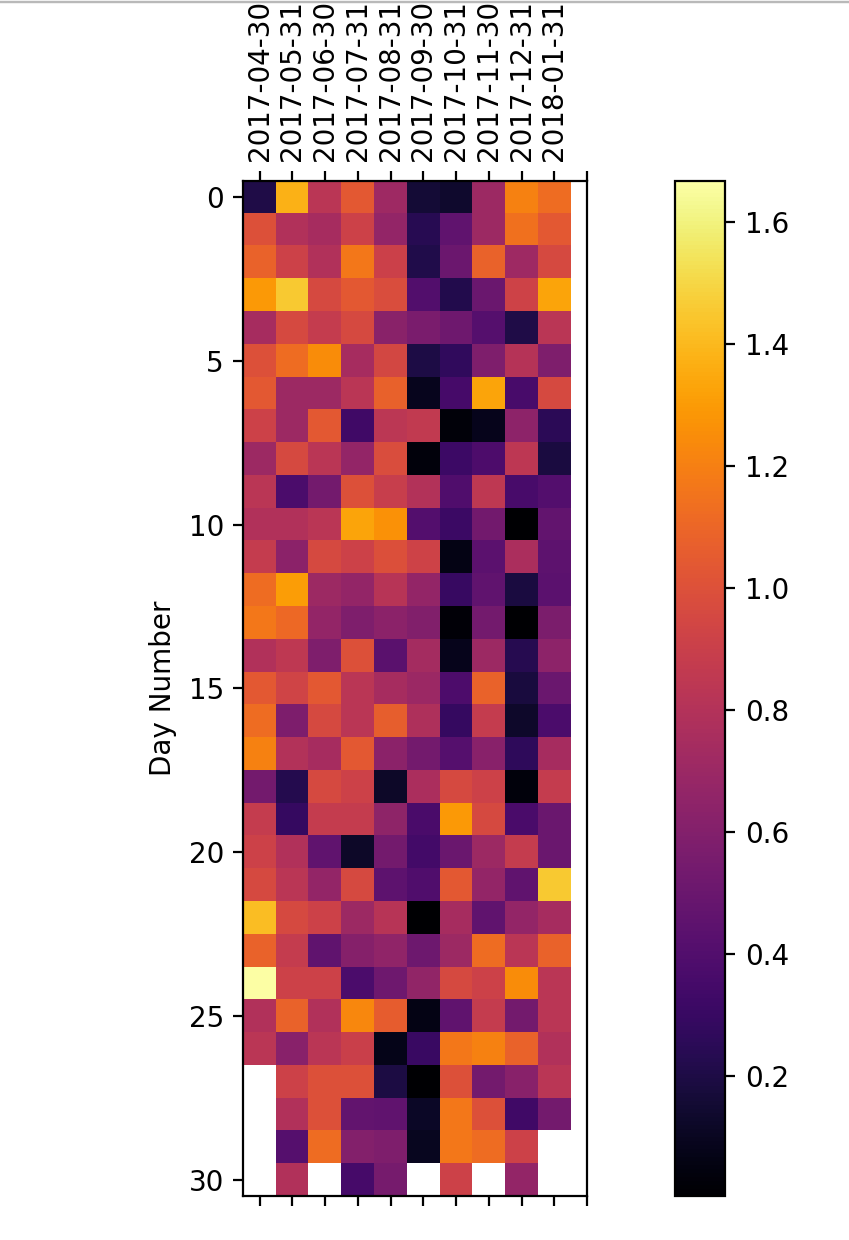


Normalized Bathroom Visits

Figure 15. Bathroom activity heatmap for subject L grouped by monthly periods.


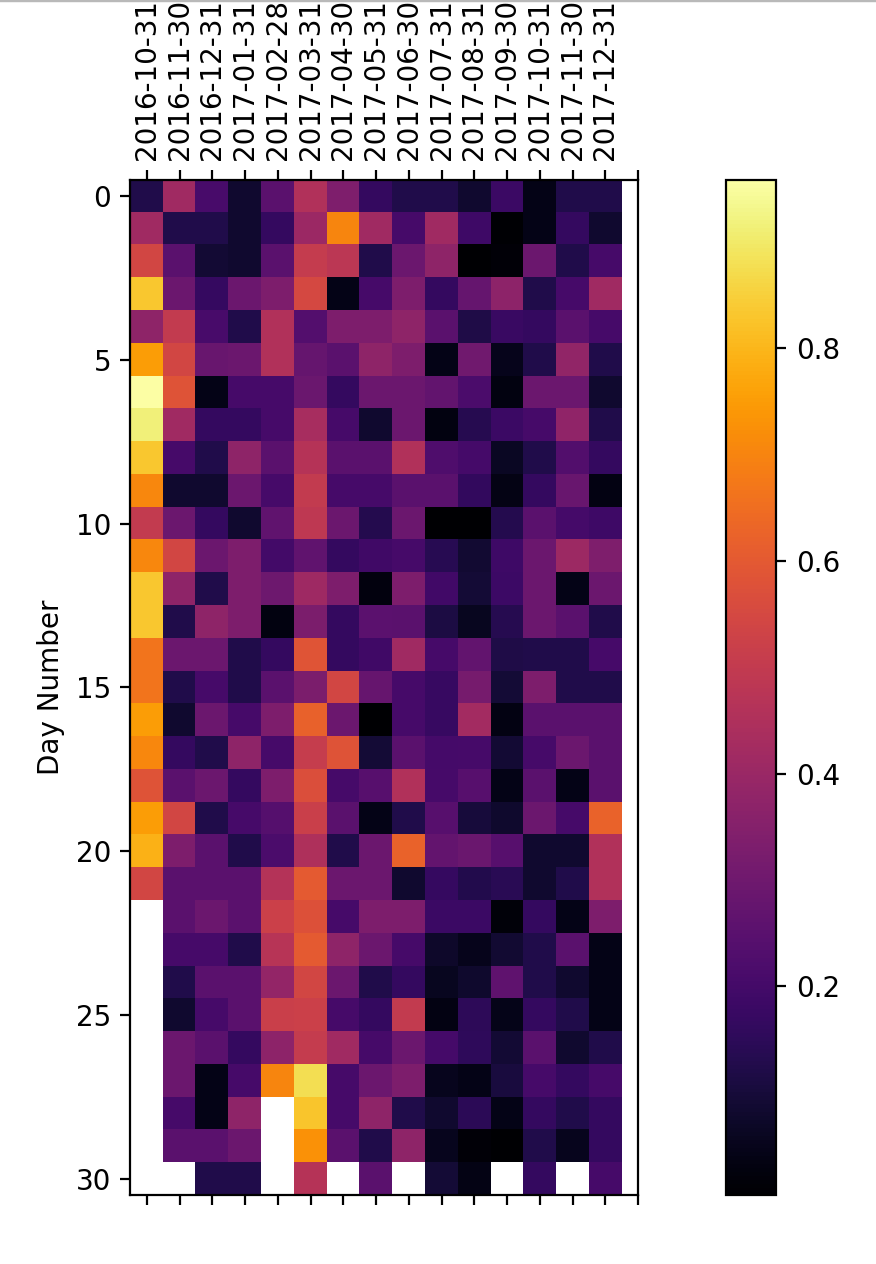


Normalized Bathroom Visits

Figure 16. Bathroom activity heatmap for subject M grouped by monthly periods.


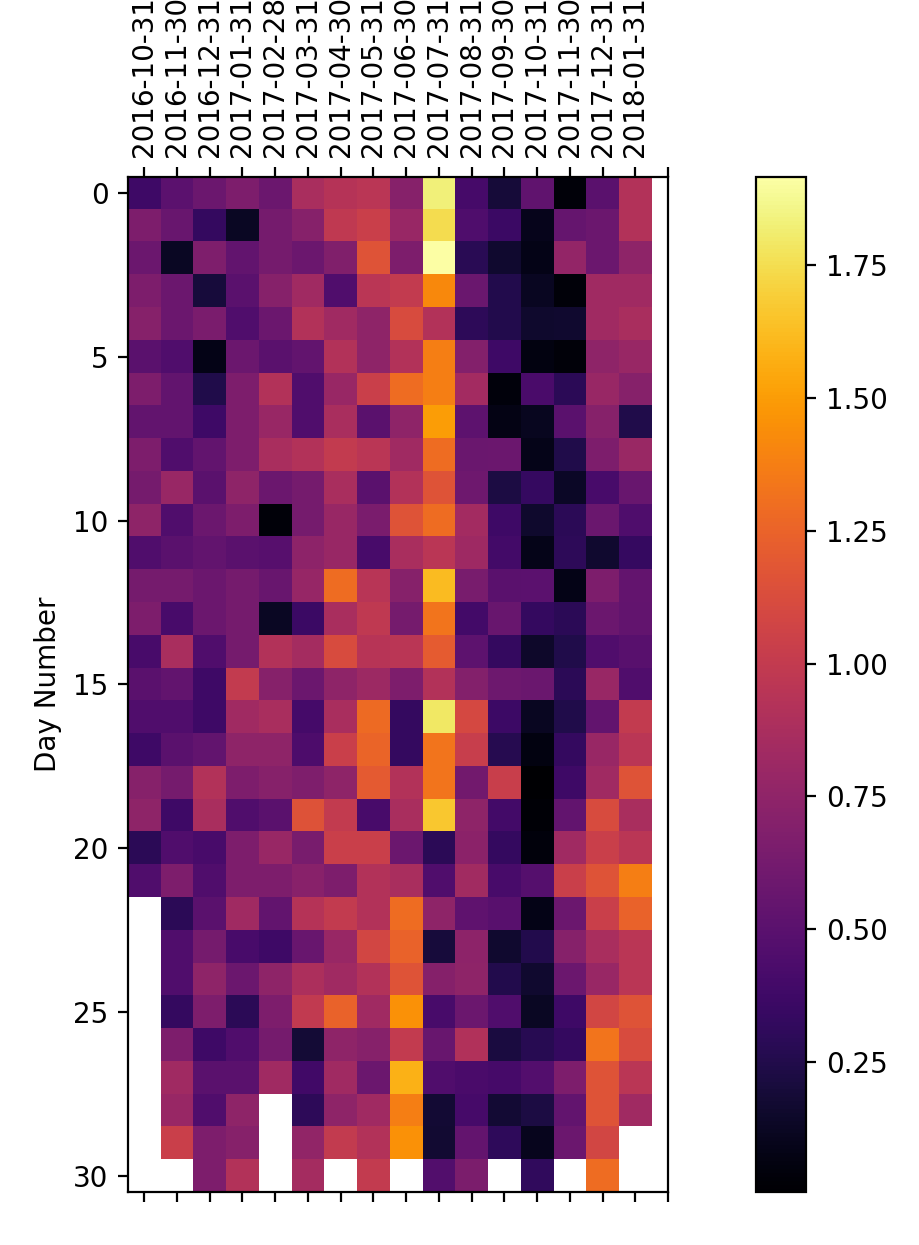


Normalized Bathroom Visits

Figure 17. Bathroom activity heatmap for subject N grouped by monthly periods.


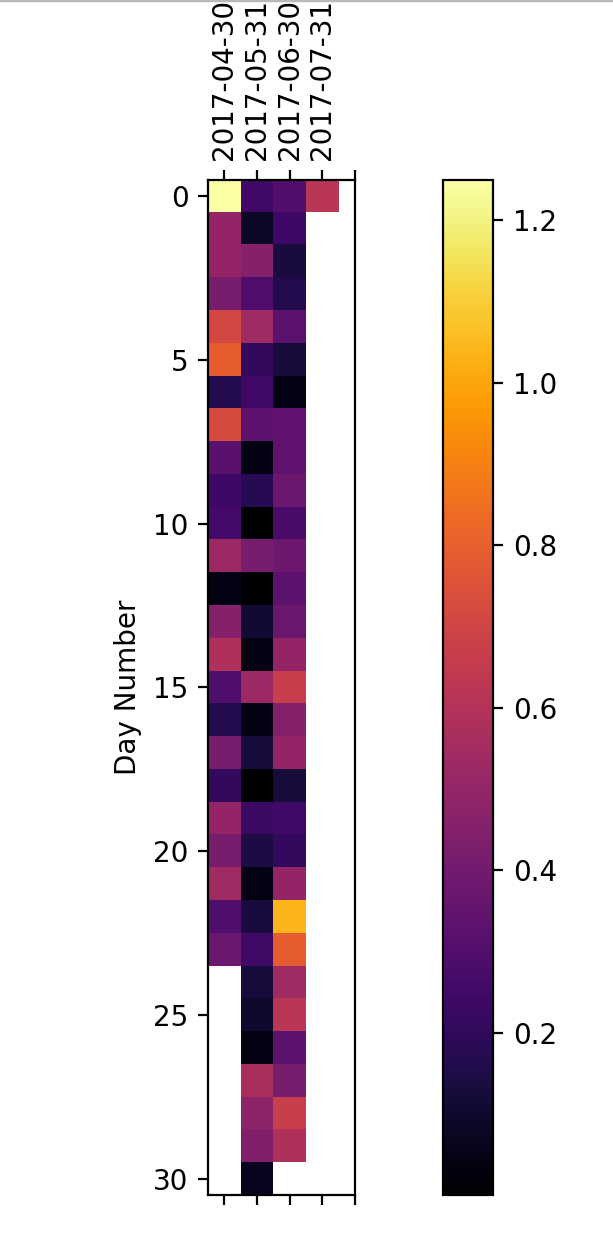


Normalized Bathroom Visits

Figure 18. Bathroom activity heatmap for subject P grouped by monthly periods.


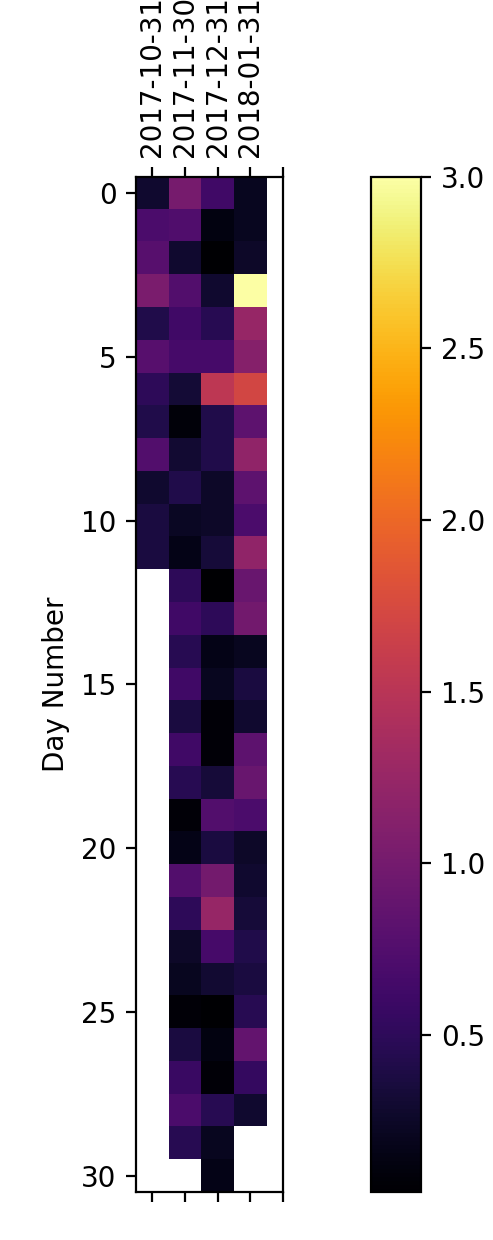


Normalized Bathroom Visits

Figure 19. Bathroom activity heatmap for subject V grouped by monthly periods.
